# Mechanistic role of metal-responsive transcription factor-1 (MTF1) in cadmium-induced prostate carcinogenesis

**DOI:** 10.7150/ijbs.110174

**Published:** 2025-05-27

**Authors:** Balaji Chandrasekaran, Bhawna Tyagi, Ashish Tyagi, Vaibhav Shukla, Mollie Schatz, Thulasidharan Nair Devanarayanan, Neha Tyagi, Chendil Damodaran

**Affiliations:** College of Pharmacy, Texas A&M University, College Station, TX, USA

**Keywords:** BPH1, MTF1, ZIC2, cadmium, metal, transformation.

## Abstract

Our previous report emphasized that chronic exposure to cadmium (10 µM) over one year led to the transformation of benign prostatic hyperplasia (BPH1) cells into malignancy through the ZIC2 signaling pathway (cerebellar zinc pathway). However, the upstream mechanisms that trigger this transformation have yet to be fully elucidated. The present study suggests that cadmium exposure induces metal regulatory element-binding transcription factor-1 (MTF1), which activates ZIC2 in BPH1 cells. Interestingly, knocking out ZIC2 expression did not affect MTF1 levels, indicating that MTF1 acts upstream of the ZIC2 signaling pathway. To further investigate the MTF-1/ZIC2 relationship, we overexpressed MTF-1 in untransformed BPH1 cells leading to the induction of ZIC2 along with other stem cell markers, such as ALDH1A1, Nanog, and CD44. This overexpression also facilitated spheroid formation. Conversely, silencing MTF1 expression in transformed cells inhibited spheroid formation and also reduced survival rate. It diminished the expression of stem cell and epithelial-to-mesenchymal transition markers and tumor growth in nude mice. Transcriptomic analysis of MTF1 silenced xenograft tumors confirmed these findings. Using CRISPR-Cas9 to knock out ZIC2 also prevented tumor formation in nude mice. These results emphasize the critical role of MTF1 in the oncogenic process and its involvement in the ZIC2-mediated transformation associated with Cd-induced malignant changes.

## Introduction

Benign prostatic hyperplasia (BPH) and prostate cancer are common diseases affecting the prostate, posing a significant burden on both patients and healthcare systems 1. Clinical and epidemiological studies have indicated that the associations and risk factors for BPH and prostate cancer are similar 2, 3. Research suggests that men with BPH may have an increased risk of developing prostate cancer 4-8 however, the specific causes, shared risk factors and underlying mechanisms remain unclear. Cadmium is a widely recognized toxic metal and a known carcinogen 9, 10. Previous research from our laboratory and others has shown that exposure to cadmium can lead to the development of prostate cancer. 11-13. Nonetheless, the precise signaling pathways through which cadmium exposure contributes to transforming benign prostatic hyperplasia into a malignant form are still not fully understood.

Metal transcription factor 1 (MTF1) has been shown to correlate with several human diseases, particularly cancers 14, 15. MTF1 regulates intracellular metal homeostasis and protects cells from excessive metal toxicity 16. Under normal conditions, MTF1 is present in the nucleus and the cytoplasm. However, it accumulates in the nucleus when heavy metal levels are elevated 17. Additionally, MTF1 translocate to the nucleus, activating downstream genes and contributing to tumor initiation and progression 18. MTF1 has potential as a diagnostic and prognostic marker for gastric and lung cancers, with a favorable prognosis associated with increased levels 19. Furthermore, MTF1 exhibits oncogenic effects and stimulates epithelial-mesenchymal transition (EMT), which lead to metastasis in ovarian tumors 20. A recent study suggests that inhibiting MTF1 reduces cell growth and increases reactive oxygen species (ROS) levels, leading to enhanced cell death in liver cancer 14. In hepatocellular carcinoma (HCC), MTF1 may represent a potential therapeutic target to modulate metal and redox balance, opening up new avenues for cancer treatment 16. However, the role of MTF1 in prostate cancer remains largely uninvestigated.

Cerebellar zinc finger 2 (ZIC2) is a transcription factor significantly expressed in solid tumors 21, 22. Elevated levels of ZIC2 are strongly associated with carcinogenesis and the self-renewal of cancer cells 23. ZIC2 facilitates the progression and metastasis of colorectal cancer by activating the TGF-β signaling pathway 24. ZIC2 overexpression is closely linked to cancer cell invasion, metastasis, and self-renewal 25. ZIC2 is crucial for maintaining tumorigenic phenotypes in EOC (epithelial ovarian cancer) cells. 22. Furthermore, silencing ZIC2 can impede EMT 26. Previous studies demonstrated that ZIC2 and markers of the Sonic hedgehog (Shh) signaling pathway, such as GLI1 and Shh, were activated in cadmium-transformed cells. This activation induced stem cell and EMT markers, including SOX2, Nanog, CD44, N-cadherin, Slug, and β-catenin upon the transformation of cadmium-exposed BPH1 cells. However, it remains unclear if MTF1 is required explicitly for ZIC2 induction and thus regulates the activation of the Shh signaling pathway, EMT, and stem cell renewal processes involved in cadmium's transformation of BPH1 cells into malignant cells.

In this study, we investigated the role of MTF1 in the cadmium-induced malignant transformation of BPH1 cells and the underlying molecular mechanisms, primarily focusing on its regulation of ZIC2 and the activation of oncogenic signaling pathways. Our results indicate that MTF1 regulates ZIC2 and its functions during the cadmium transformation of BPH1 cells. In mouse models, the knockdown of MTF1 and the knockout of ZIC2 significantly inhibited tumor growth of cadmium-transformed BPH1 (CTBPH1) cells, suggesting a potential therapeutic strategy for prostate cancer.

## Materials and Methods

### Cell lines and reagents

Dr. Simon W. Hayward (Northshore Research Institute, University of Chicago, Pritzker School of Medicine) kindly provided the BPH1 cell line. After one year of treatment with 10 µM cadmium (Sigma, Dallas, TX, USA), the cells transformed into a malignant phenotype. The transformed cells were designated as cadmium-transformed BPH1 cells (CTBPH1) 27. BPH1 and CTBPH1 cells were grown in RPMI medium containing 10% fetal bovine serum, 1% antibiotic and antimycotic solution, and a humidified atmosphere of 5% CO_2_ at 37 °C in an incubator. LOR-253 is an MTF1 inhibitor purchased from AdooQ Bioscience (A13838).

### Viability assay

The effect of MTF1 shRNA was determined using the trypan blue viability assay. Cells were plated on 6-well plates 27. Cells were then enriched and transfected with MTF1 shRNA and the corresponding control. After transfection and cadmium exposure, cells were harvested and stained with an equivalent volume of 0.4% trypan blue dye for one minute. A hemocytometer was utilized to enumerate dead cells.

### shRNA transfection and overexpression

BPH1 and CTBPH1 cells were grown in six-well plates at a density of 3 × 10^5^ cells per well. Cells were enriched and then transiently transfected with MTF1(Origene:TR311365) or ZIC2-specific short hairpin RNA (shRNA; Origene: TR308270); control shRNA; or MTF1 (SC116396) and ZIC2 overexpression plasmids (SC303882) for 48 hours. Cells were then harvested after being treated with either vehicle or cadmium. Lipofectamine® 2000 was utilized as the transfection agent. Whole-cell lysates of transfected BPH1 and CTBPH1 were also prepared for western blot analysis after treatment with vehicle or cadmium. Transfected cells were harvested after 48 hours and utilized for cell proliferation, colony formation, sphere formation, invasion, and migration assays. OriGene Technologies Inc. provided MTF1 overexpression and ZIC2 overexpression plasmids and MTF1 and ZIC2 shRNAs.

### Sphere formation assay

BPH1 and CTBPH1 cells were seeded in six-well plates at a density of 3 × 10^5^ cells/well. After 24 h, cells were transiently transfected with ZIC2, MTF1, or control shRNA, or ZIC2 or MTF1 overexpression plasmid for 48 h. Lipofectamine® 2000 was used as the transfection reagent. After 48 h, the single cell suspension (2 x 10^3^ cells in 200 μL) of the appropriate culture medium supplemented with Matrigel (354254, Corning) was loaded into each well of round-bottom ultra-low attachment plates. The cell growth morphology of the spheroids was then characterized in triplicate over a 7-day culture period.

### Invasion assay

A 24-well tumor invasion system (BD BioSciences, San Jose, CA) was used to inoculate BPH1, CTBPH1, and MTF1 knockdown, MTF1 overexpression, and corresponding control cells in serum-free DMEM on Matrigel pre-coated inserts (BD BioCoatTM). As a chemoattractant, DMEM supplemented with 10% FBS was added to the invasion system of the lower chamber for 24 h. After 20 min of methanol preservation, the Transwell inserts were stained with crystal violet for 5 min. Invading cells from at least three distinct areas were counted in images taken at 10x magnification. 28

### Wound healing assay

After seeding cells on 6-well plates, a single scratch was made across the monolayer with a 100 μL pipette tip once cells reached 80-90% confluence. After gently washing the plate with medium to remove any nonadherent cells, a new RPMI was added to each well. Photomicrographs were taken at 0 and 24 h following the scratch. Migration in the wound area was examined using ImageJ software.

### Western blot and Immunoprecipitation

Western blot and immunoprecipitation assays were performed in BPH1 and CTBPH1 cells, which were lysed in RIPA lysis buffer (Thermo Scientific, Rockford, IL, USA) supplemented with a cocktail protease inhibitor (Sigma, Dallas, TX, USA). The proteins were separated by SDS-PAGE and transferred to polyvinylidene difluoride membranes (Bio-Rad, USA). Membranes were blocked in Bio-Rad blocking buffer for 10 min at room temperature and incubated with primary antibodies overnight at 4 °C, followed by a 1 h incubation with HRP-conjugated secondary antibodies.

Western blotting was performed using specific antibodies against MTF1 (ab236401), ZIC2 (ab150404), ZIC2 (immunohistochemistry) (Sigma AU35821), Shh (ab53281), PARP (CST, #9542), NFκB p65 (CST, #8242), Bcl-2 (CST, #15071), Bcl-xL (CST, #2764), X-linked inhibitor of apoptosis protein (XIAP; Cell signaling #2045), ALDH1A1 (D4R9V) (CST, #12035), SOX2 (CST, #3579), NANOG (cell signaling #3608), CD44 (cell signaling #5604), Slug (CST, #9585), E-Cadherin (CST, #3195), β-catenin (CST, #8480), N-cadherin (CST, #13116), or β-Actin (CST, #4970; loading control). Total cell lysates were treated with an anti-ZIC2 antibody or the corresponding IgG control on a rotating shaker at 4 °C overnight. The proteins were then immunoprecipitated using protein A/G agarose (Sigma P2055). The beads were collected, and a loading buffer was added to the boiling solution. The supernatants were then detected by immunoblotting. Protein bands were visualized using the Bio-Rad ChemiDocTM imaging system.

### Promoter assay

BPH1 and CTBPH1 cells (3× 10^5^ cells) were plated in a six-well plate. After 24 hours, cells were transfected with MTF1 reporter or corresponding controls with Lipofectamine® 2000 transfection reagent. After transfection, cells were treated with cadmium, we used an MRE (Metal Regulatory Elements) reporter plasmid, as MTF-1, also known as the MRE-binding transcription factor-1, directly interacts with MREs to activate gene transcription. The promoter activity was estimated using the Dual-Glo Luciferase Assay System (Promega, Madison, WI), and cells were processed as per the manufacturer's protocol.

Seven putative MTF1 binding sites in the ZIC2 promoter were identified. The sites with stronger affinity, as determined *in silico*, were amplified and cloned into the pRMT-Luc promoter-less Firefly Luciferase Reporter Vector (Origene) using EcoR1/Xho1 and Nhe1/Mlu1 restriction enzymes (Promega). Clone1: -1580 to -1573, -1559 to -1551, -1370 to -1363, and -1226 to -1219. Clone 2: -605 to -597 and Clone 3: -202 to -188. The T4 ligase was purchased from Takara. For the extraction and purification of the constructed reporter plasmids, we used a plasmid miniprep reagent kit (Qiagen), and S.N.A.P. gel purification kit (Invitrogen) for plasmid DNA isolation and DNA purification. Next, we transfected the pRMT-luc vector containing the MTF1 binding sites into MTF1-overexpressed BPH1 cells using Lipofectamine 2000 and assessed the luciferase activity.

### Chromatin immunoprecipitation (ChIP)

The ChIP-Seq High Sensitivity Kit (Abcam, Cambridge, UK, ab185908) was used in this experiment. The CTBPH1 cells are fixed with formaldehyde to extract the chromatin and then lysed. The immunological complex was generated by adding the MTF1 antibody after shearing the chromatin. The DNA was then released and purified after the complexes were pelleted. Finally, quantitative real-time PCR (qRT-PCR) was used to experiment. Primer sequences: FP: CTCTAGTTACCTCGGGAGCG. RP: GGAGAAGAAGCCGTCAGACA.

### Real-time quantitative PCR (RT-qPCR)

Total RNA was extracted from BPH1 and CTBPH1 cells (2, 4, 6, 8, 10, and 12 months) according to the Qiagen protocol, and cDNA was synthesized using 2 μg of total RNA with iScript Reverse Transcription Supermix (Bio-Rad, Hercules, CA, USA) according to the manufacturer's protocol. Advanced Universal SYBR Green Supermix (Bio-Rad, Hercules, CA, USA) was used to measure gene expression. The endogenous control was β-actin. We used the CFX-connect real-time system (Bio-Rad, Hercules, CA, USA) to perform real-time PCR for MTF1 expression. MTF1 primer sequences are FP: CAGTGAGGAGAACACTTGC. RP: TGCACATAACCCTGAGACATT. β-actin primer sequences are FP: ATGATCTGGGTCATCTTCTC. RP: GACGACATGGAGAAAATCTG.

### RNA seq

A total RNA isolation system (Promega, USA) was used to isolate total RNA from xenograft tumors. We enriched the isolated RNA for mRNA using a QuantSeq 3' mRNA-seq library prep kit (Lexogen, Austria), following cDNA conversion and library preparation. We used an Illumina NextSeq500 platform (CA, USA) for the sequencing.

### Functional enrichment analysis

GSEA software version 4.2.2 was used for gene set enrichment analysis (GSEA). We generated a Principal Component Analysis plot using the ClusVis web tool described previously 29 and heat maps for volcano plots were produced using SRplot (https://www.bioinformatics.com.cn/en), a free online platform for data analysis and visualization.

### Immunofluorescence and Immunohistochemistry analysis

Eight-chamber glass slides were used to seed BPH1 and CTBPH1 cells. After fixation and labeling of cells with MTF1 and ZIC2 antibodies, secondary antibodies were added to determine expression and localization. A KEYENCE BZ-X800 series fluorescence microscope was used to examine the cells. Xenograft tumor tissues were embedded in paraffin, fixed, and cut into thin slices (approximately 4μm thick). After xylene and alcohol dewaxing, xylene was removed from these sections using an alcohol concentration gradient of 90%, 80%, and 70%. The following steps were antigen retrieval and deactivation of endogenous peroxidase. After overnight incubation at 4 °C with tumor sections, primary antibodies against MTF1, ZIC2, and Ki67 were applied individually. The secondary antibodies were added, and the mixture was incubated for one hour at room temperature. After adding a new DAB solution, the staining results can be observed and captured under a microscope.

### CRISPR/Cas9 mediated gene knockout

We performed CRISPR/Cas9-mediated gene knockout in CTBPH1 cells for ZIC2 according to the manufacturer's protocol, Origene (KN420798). Briefly, to achieve 50-70% confluence, we plated 200,000 cells in 2 mL of growth media in each well of a 6-well plate around 18 to 24 hours prior to transfection. For each transfection, 1 μg of gRNA vector (ZIC2 gRNA vector 1 Target Sequence: TTCACGGTCCTGCATCTCGG; ZIC2 gRNA vector 2 Target Sequence: CTTGAAGGCTCCCATGTGCG) and 1 μg of donor DNA (LoxP-EF1A-tGFP-P2A-Puro-LoxP) were diluted in 250 μL Opti-MEM Media. After vortexing, 6 μL of turbofectin was added to the DNA mix and incubated for 15 minutes at room temperature. The transfection mixture was added dropwise to the cells without changing the media. After incubation in a 5% CO2 incubator, cells were split 1:10 at 48 hours post-transfection and grown for 3 additional days before repeating the split. Cells were split 4 times to allow dilution of episomal DNA before puromycin selection. Cells were maintained in the RPMI media and passaged every 2-3 days. Selection was continued for 4 splits and knockout was confirmed with western blot with ZIC2 antibody (ab150404).

### Xenograft Study

The Animal Care and Use Committee of Texas A&M University approved the study protocol on tumorigenesis of athymic mice. Five-week-old male BALB/c (nu/nu) nude mice were purchased from the Jackson Laboratory and maintained in a pathogen-free environment in the vivarium at Texas A&M University. The mice were randomly divided into five groups, six mice each. After a week of acclimatization, the nude mice received subcutaneous injections of CTBPH1, stable MTF1 knockdown cells, and stable ZIC2 KO cells (1 × 10^6^ cells in 100 μL of PBS with Matrigel). We started tumor size measurement (twice a week) once control CTBPH1 tumor sizes reached 50 mm³. At the end of the experiment, tumors were harvested and weighed, and their volume was measured. The tumors were utilized for RNA sequencing analysis and histopathology studies.

### Statistical analysis

Cell culture experiments are performed in triplicates. The mean differences between the two groups were determined using a two-tailed Student's t-test. A p-value less than 0.05 is considered significant. Data are presented as mean ± standard deviation (SD).

## Results

### Chronic cadmium exposure activates the MTF1 and ZIC2 signaling pathways in BPH1 cells

**Figure 1:**
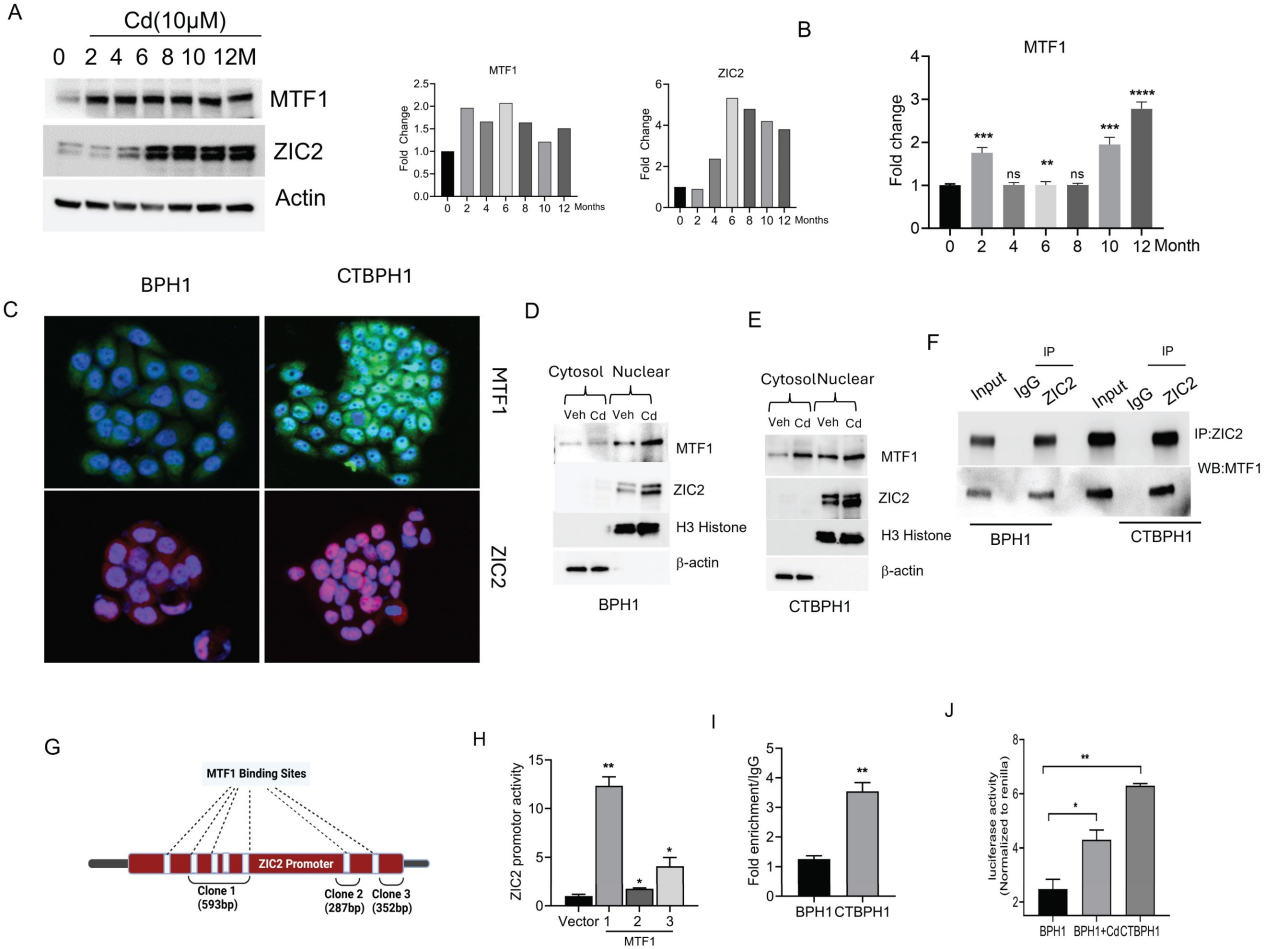
**Chronic cadmium exposure activates the MTF1 and ZIC2 signaling pathways in BPH1 cells. A**. An Immunoblot of MTF1, ZIC2 and β -actin in BPH1 cells was treated with 10 µm cadmium from 0 to 12 months. **B**. qRT-PCR of indicated mRNA in BPH1 cells treated with 10µm cadmium from 0 to 12 months. **C**. Immunofluorescence expressions of MTF1 and ZIC2 in BPH1 and CTBPH1 cells. **D&E**. Immunoblots of cytosolic and nuclear fraction for the expressions of MTF1 and ZIC2 in BPH1 and CTBPH1 cells. **F**. BPH1 and CTBPH1 cell lysates were immunoprecipitated with ZIC2 antibody. Immunoblot analysis was performed on the immunoprecipitates using the anti-MTF1 antibody. **G**. Direct binding of MTF1 to the ZIC2 promoter region shows up: Three luciferase reports that include different DNA sequences of the ZIC2 promoter region are displayed in the schematic diagram. **H**. pRMT vector cloned with MTF1 binding sites in the ZIC2 promoter transfected with MTF1 overexpressed BPH1 cells for ZIC2 promoter activity. **I**. ChIP was performed using chromatin prepared from BPH1 and CTBPH1 cells with MTF1 antibody, and enriched DNA was quantified using qRT-PCR and ZIC2 primers. **J**. MRE luciferase reporter was transfected into BPH1, CTBPH1, and cadmium-exposed BPH1 cells to measure luciferase activity.

We previously reported that chronic exposure to cadmium (10 μM) could transform normal BPH1 cells into malignant cells (CTBPH1) by activating ZIC2 signaling in 12 months. We performed a western blot analysis to gain molecular insights into the transformation of BPH1 cells. Results suggested that cadmium significantly induces the expression of MTF1 as early as 2 months post-exposure, with subsequent induction of ZIC2 observed at 6 months in BPH1 cells (Fig. 1A). We further validated the transcripts of MTF1 expression through quantitative real-time polymerase reaction (qRT-PCR) (Fig. 1B). Further, nuclear localization of MTF1 and ZIC2 were confirmed by immunofluorescence assays (Fig. 1C). To confirm the nuclear localization of MTF1 and ZIC2, we conducted western blot analysis on nuclear and cytoplasmic extracts from both BPH1 and CTBPH1 cells cadmium increased MTF1 and ZIC2 accumulation in the nuclei as compared to the vehicle-treated cells (Fig. 1D and E). The immunoprecipitation with ZIC2 and subsequent western blot analysis with MTF1 confirmed the interaction between these two proteins in transformed BPH1 cells (Fig. 1F).

We investigated whether MTF1 binds to the ZIC2 promoter through an in-silico analysis and identified seven potential MTF1 binding sites within the ZIC2 promoter region (Fig. 1G).

The sites are: -1580 to -1573, -1559 to -1551, -1370 to -1363, -1226 to -1219, -605 to -597, -202 to -188. These fragments were amplified and cloned (clone 1, 2 and 3) into the pRMT-Luc Promoter-less Firefly Luciferase Reporter Vector. As shown in Fig. 1H, clone-1, which has four binding sites, significantly enhanced the activity of the ZIC2 promoter. These results suggest that MTF1 regulates and activates ZIC2 in cadmium transformed cells. To further confirm these results, we performed chromatin immunoprecipitation (ChIP) assays, confirming MTF1's binding to the ZIC2 promoter (Fig 1I). Subsequently, we sought to determine whether cadmium exposure enhances MTF1 activity in BPH1 cells. To achieve this, we employed an MRE (Metal Regulatory Elements) reporter plasmid, MTF-1 is also referred to as MRE-binding transcription factor-1 as it directly interacts with MREs to activate gene transcription. Therefore, an increase in MTF1 activity would correlate with heightened MRE activation. As expected, we observed a rise in luciferase activity in BPH1 cells following exposure to cadmium and also in CTBPH1 cells (Fig 1J). These results suggest that MTF1 interacts with and transcriptionally activates ZIC2 in transformed BPH1 cells.

### Silencing the MTF1 gene impedes stemness, proliferation, and EMT in CTBPH1 cells

**Figure 2:**
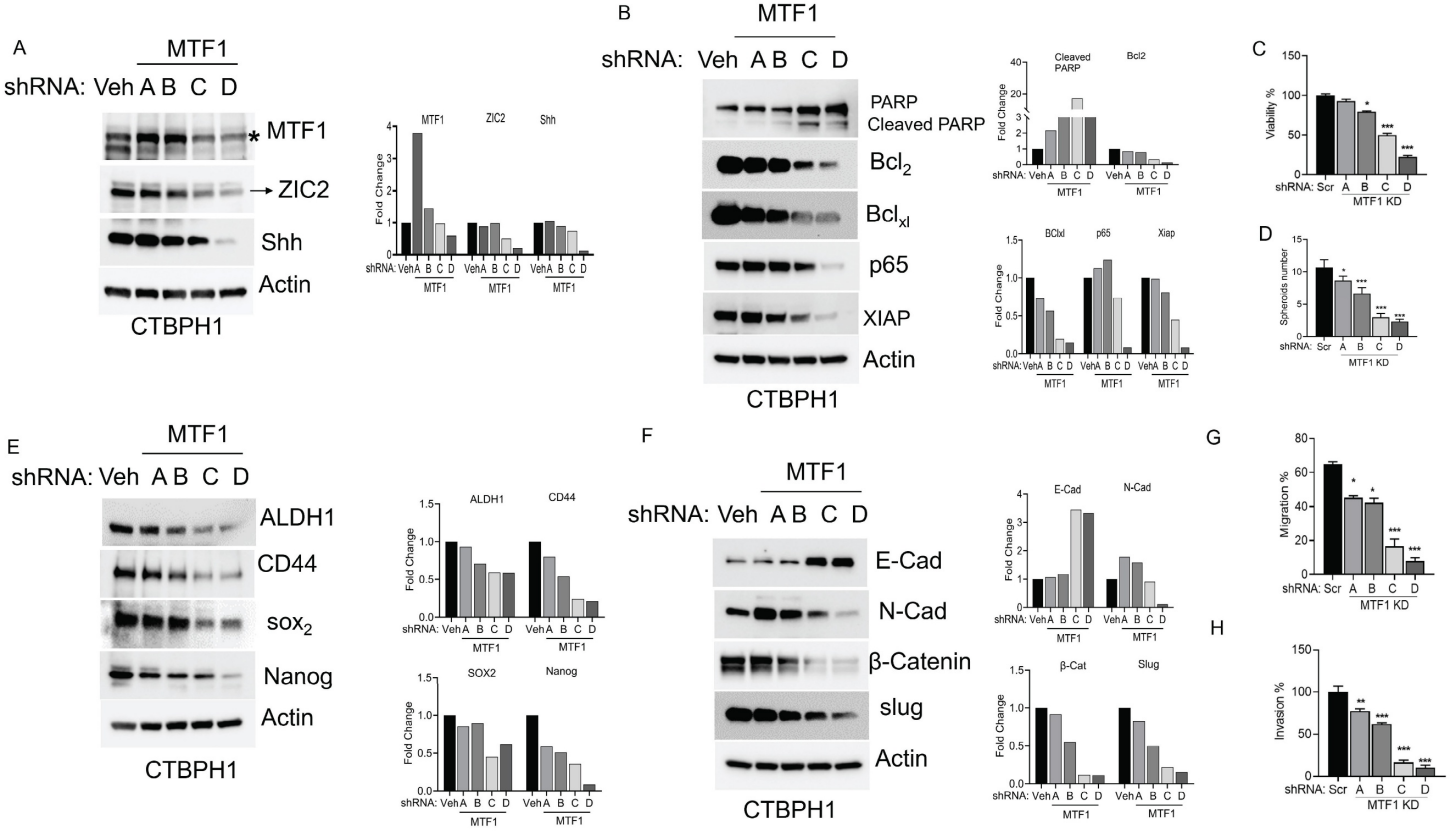
**Silencing the MTF1 gene impedes stemness, proliferation, and EMT in CTBPH1 cells. A**. An Immunoblot of MTF1, ZIC2, and Shh and β-actin in shRNA MTF1(4 unique shRNAs) knockdown CTBPH1 cells. **B**. Immunoblot of PARP, Bcl2, Bclxl, p65, XIAP, and β-actin in shRNAMTF1(4 unique shRNAs) knockdown CTBPH1 cells. **C**. Cell viability of shRNA MTF1(4 unique shRNAs) knockdown in CTBPH1 cells. **D**. Spheroid assay of shRNA MTF1(4 unique shRNAs) knockdown in CTBPH1 cells. **E.** Immunoblot of stem cell markers such as ALDH1A1, CD44, SOX2, Nanog, and β-actin shRNAMTF1(4 unique shRNAs) knockdown CTBPH1 cells. **F**. Immunoblot of EMT markers such as E-cadherin, N-Cadherin, β-catenin, Slug, and β-actin in shRNA MTF1(4 unique shRNAs) knockdown CTBPH1 cells. **G&H**. MTF1 knockdown CTBPH1 cells failed to promote the migration and invasion of CTBPH1 cells compared to control by wound healing and invasion assays.

To investigate the function of the MTF1 gene in CTBPH1 cells, we used specific shRNAs to knockdown MTF1 expression, which resulted in the downregulation of ZIC2 expression, as confirmed by western blot analysis (Fig 2A). Subsequent analyses of pro-survival and pro-apoptotic proteins revealed that MTF1 knockdown increased the expression of cleaved PARP and decreased levels of key survival markers, including Bcl2, Bcl-xL, p65, and XIAP (Fig 2B). To assess the impact on cell proliferation, we performed cell proliferation assays, which showed a significant reduction in cell proliferation in MTF1-silenced cells compared to the control group (Fig 2C). Previously, we demonstrated that CTBPH1 cells can form spheres under ultra-low attachment conditions; however, the knockdown of MTF1 impaired sphere formation in these cells. (Fig 2D). Notably, the depletion of MTF1 reduced the expression of stem cell markers such as ALDH1A1, CD44, SOX2, and Nanog (Fig 2E).

Additionally, MTF1 silencing increased the expression of the epithelial cell marker E-cadherin while decreasing mesenchymal markers such as N-cadherin, β-catenin, and Slug (Fig 2F). This suggests a shift towards an epithelial phenotype. We examined cell migration using a wound-healing assay to evaluate how MTF1 influences cell migration in CTBPH1 cells. The results indicated significantly reduced migration in MTF1 knockdown cells compared to control CTBPH1 cells (Fig 2G). Furthermore, a transwell assay with Matrigel-coated inserts revealed a significant reduction in cell invasion in MTF1 knockdown cells compared to control cells (Fig 2H). These findings elucidate a novel molecular mechanism by which MTF1 promotes EMT. This mechanism involves MTF1 decreasing ZIC2 expression and attenuating stem cell markers and cell survival pathways which diminish EMT transition in CTBPH1 cells.

### Cadmium fails to restore ZIC2 function in MTF1 knockdown CTBPH1 cells

**Figure 3:**
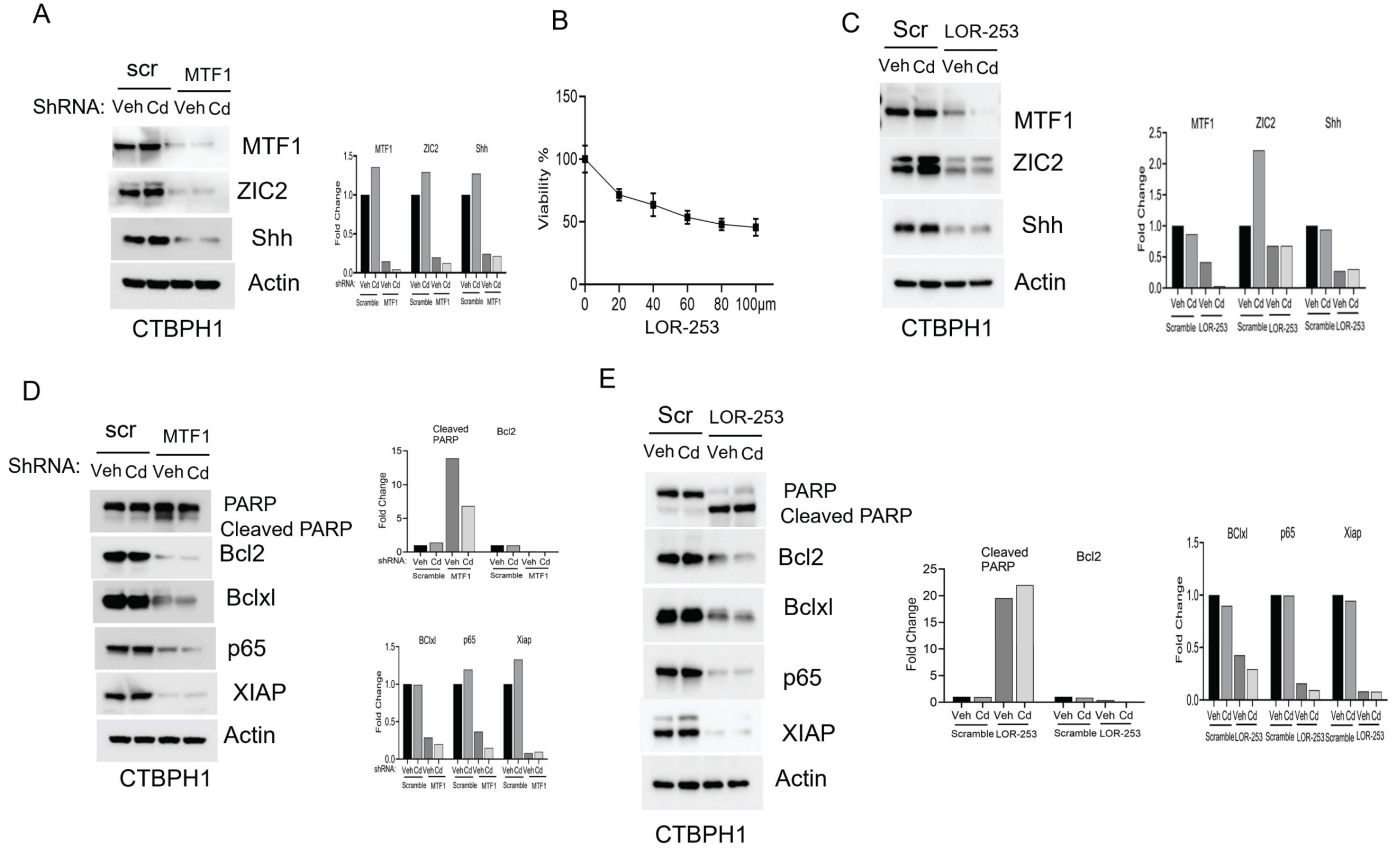
**Effect of shRNA MTF1 knockdown or pharmacological inhibitor of MTF1 (LOR-253) in cadmium exposed CTBPH1 cells. A**. An Immunoblot of MTF1, ZIC2, and Shh and β-actin in Cd-exposed shRNA MTF1 knockdown CTBPH1 cells. **B**. Cell viability of CTBPH1 cells treated with different concentrations of LOR-253 for 24h. **C.** Immunoblot of MTF1, ZIC2, and Shh and β-actin in cadmium exposed LOR-2 CTBPH1 cells.** D**. Immunoblot of PARP, Bcl2, Bclxl, p65, XIAP, and β-actin in cadmium exposed shRNA MTF1 knockdown CTBPH1 cells. **E.** Immunoblot of PARP, Bcl2, Bclxl, p65, XIAP, and β-actin in cadmium exposed LOR-253 treated CTBPH1 cells.

To confirm that the oncogenic role of ZIC2 signaling, we silenced MTF1 using shRNA-D in CTBPH1 cells and then exposed the cells to cadmium. The knockdown of MTF1 resulted in the downregulation of ZIC2 expression, and exposure to cadmium did not restore the expression levels of MTF1 or, ZIC2 or Shh (Fig. 3A). We further inhibited MTF1 through pharmacological inhibition using LOR-253. We found that the IC50 dose of LOR-253 was 73 µM at 24 hours in CTBPH1 cells. (Fig 3B). As shown in Fig. 3C, treatment with LOR-253 led to a downregulation of both MTF1, ZIC2 and Shh expression, and again, cadmium exposure could not reverse this effect. After confirming that MTF1 silencing downregulated ZIC2 expression, we analyzed the prosurvival markers in CTBPH1 cells. MTF1 knockdown increased cleaved PARP levels while decreasing the expression of survival markers such as Bcl2, Bcl-xl, p65, and XIAP. Notably, exposure to cadmium did not restore the levels of these survival markers (Fig. 3D). Additionally, treatment with LOR-253 in CTBPH1 cells increased cleaved PARP levels. It decreased the expression of Bcl2, Bcl-xl, p65, and XIAP. (Fig. 3E). These results strongly suggest that MTF1 knockdown with cadmium exposure modulates pro-survival signaling in CTBPH1 cells.

**Figure 4:**
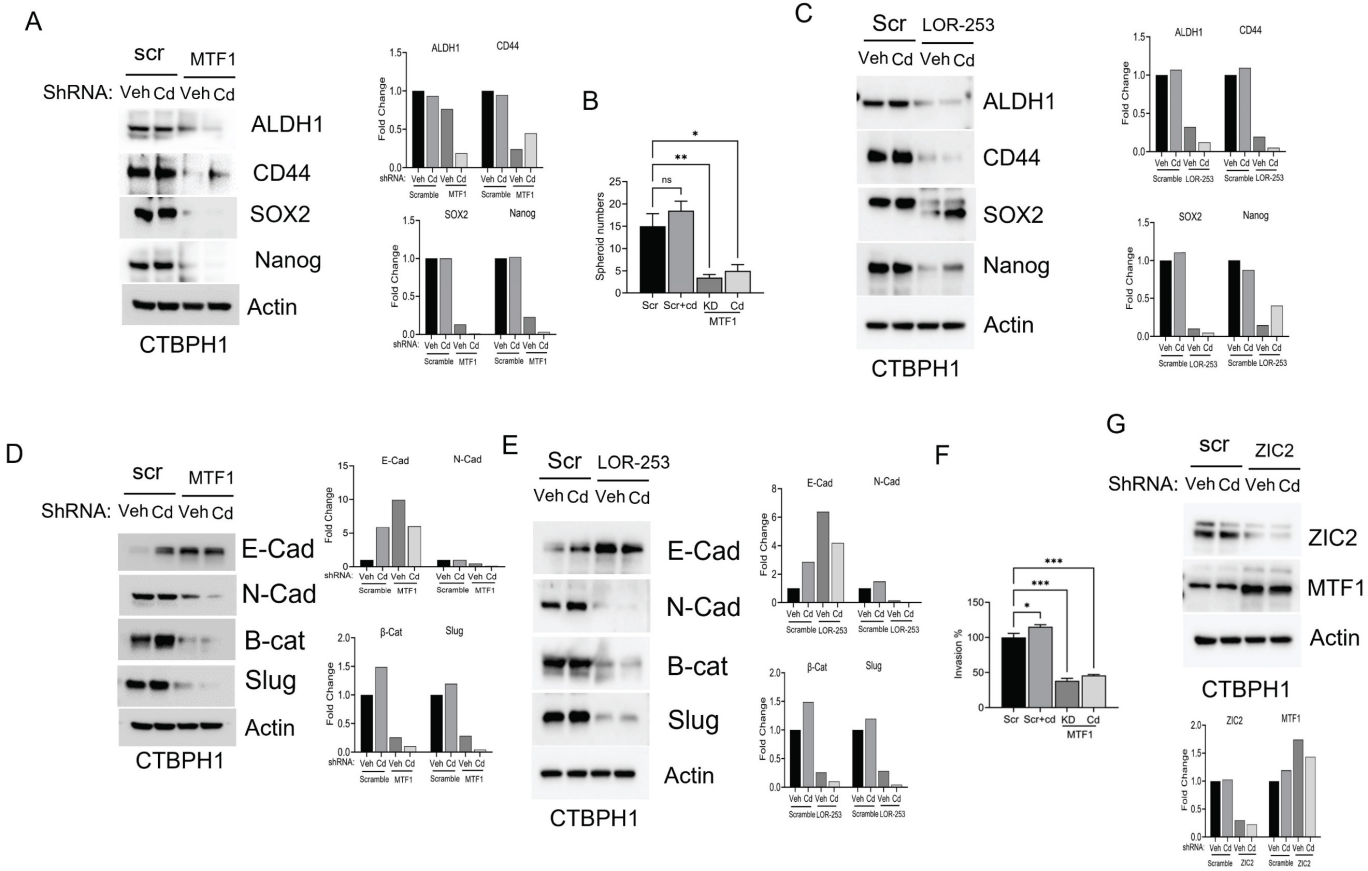
**Cadmium fails to restore the function of MTF1 and ZIC2 in CTBPH1 cells**. **A.** Immunoblot of stem cell markers such as ALDH1A1, CD44, SOX2, and Nanog and β-actin in cadmium exposed ShRNA MTF1 knockdown CTBPH1 cells. **B.** Spheroid assay for cadmium exposed ShRNA MTF1 knockdown CTBPH1 cells. **C.** Immunoblot of stem cell markers such as ALDH1A1, CD44, SOX2, and Nanog and β-actin in cadmium exposed in cadmium exposed LOR-253 treated CTBPH1 cells.** D.** Immunoblot of EMT markers such as E-cadherin, N-Cadherin, β-catenin, Slug, and β-actin in cadmium exposed shRNA MTF1 knockdown CTBPH1 cells. **E.** Immunoblot of EMT markers such as E-cadherin, N-Cadherin, β-catenin, Slug, and β-actin in cadmium exposed LOR-253 treated CTBPH1 cells. **F.** Invasion assay for cadmium exposed ShRNA MTF1 knockdown CTBPH1 cells. **G.** Immunoblot for ZIC2 and MTF1 in Cadmium exposed ZIC2 knockdown CTBPH1 cells.

Next, we examined the impact of MTF1 silencing on the expression of stem cell markers. Silencing MTF1 using shRNA and LOR-253 led to downregulating key stem cell markers, including ALDH1A1, CD44, SOX2, and Nanog. Subsequent spheroid assays demonstrated that MTF1 silencing, inspite of cadmium exposure, prevented CTBPH1 cells from forming spheroids, confirming that MTF1 expression is associated with the self-renewal properties of cadmium-exposed cells (Figs 4A-C).

Moreover, MTF1 knockdown through shRNA and LOR-253 reduced the levels of mesenchymal markers, including N-cadherin, β-catenin, and Slug, which could not be rescued even after cadmium exposure (Fig. 4D & 4E). MTF1 silencing also significantly impeded *in vitro* cell invasion in CTBPH1 cells, and cadmium exposure failed to accelerate these processes. (Fig. 4F). Interestingly, ZIC2 shRNA knockdown did not alter MTF1 expression in CTBPH1 cells (Fig. 4G), suggesting that MTF1 could be upstream of ZIC2. Overall, these findings confirm that MTF1 knockdown in CTBPH1 cells leads to the downregulation of stem cell markers through ZIC2 and mitigates the EMT transition.

### Overexpression of MTF1 or ZIC2 mimics the effects of cadmium in BPH1 cells

**Figure 5:**
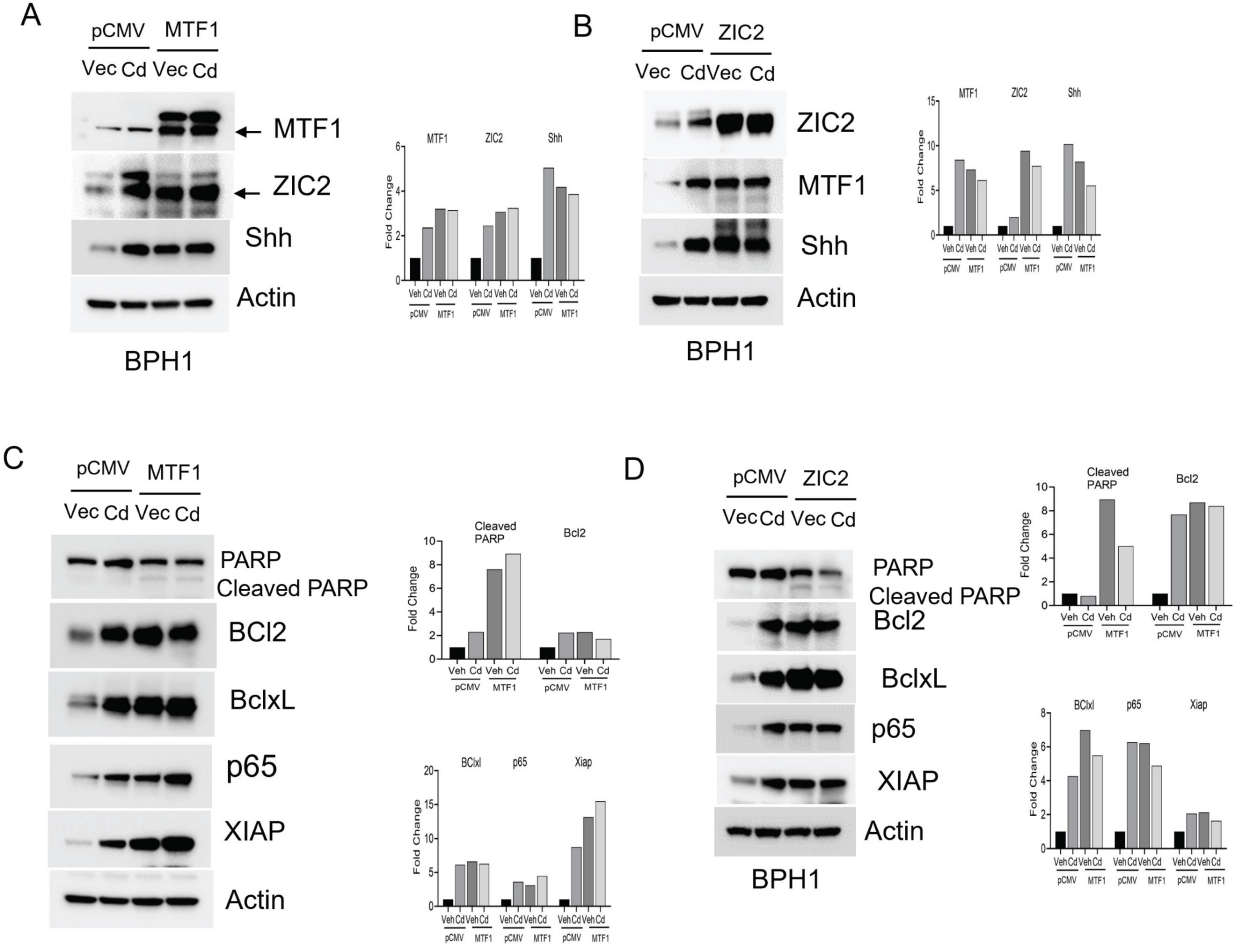
**Overexpression of MTF1 and ZIC2 with cadmium exposure activates pro-survival mechanisms in BPH1 cells. A**. Immunoblot of MTF1, ZIC2, and Shh and β-actin in cadmium exposed MTF1 overexpressed BPH1 cells. **B**. Immunoblot of MTF1, ZIC2, and Shh and β-actin in cadmium exposed ZIC2 overexpressed BPH1 cells. **C**. Immunoblot of PARP, Bcl2, Bclxl, p65, XIAP, and β-actin in MTF1 overexpressed BPH1 cells. **D.** Immunoblot of PARP, Bcl2, Bclxl, p65, XIAP and β-actin in ZIC2 overexpressed BPH1 cells.

Our studies showed that silencing or knocking out MTF1 or ZIC2 resulted in antiproliferative effects, stem changes, and activating EMT markers. To investigate the potential impact of overexpressing MTF1 or ZIC2 on cadmium, we introduced overexpression plasmids for MTF1 or ZIC2 into BPH1 cells. We found that overexpression of MTF1 led to an increase in ZIC2 expression, and conversely, overexpression of ZIC2 upregulated MTF1 expression in BPH1 cells. We used cadmium- treatment as a control for our experiments, and exposure to cadmium further elevated the expression of both MTF1 and ZIC2 in these cells (Fig. 5A &B).

**Figure 6:**
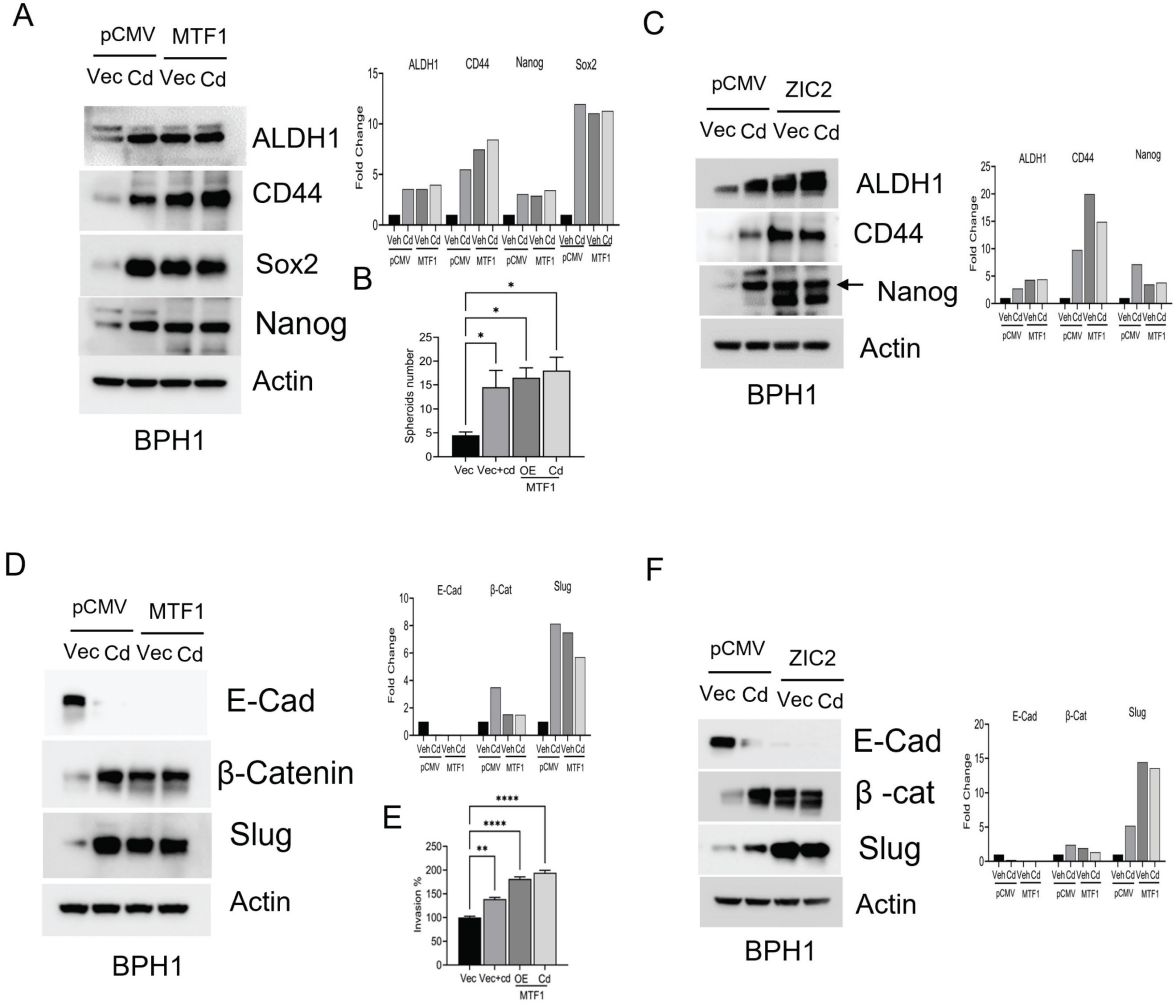
** Overexpression of MTF1 and ZIC2 mimics the effects of Cadmium in BPH1 cells. A**. Immunoblot of stem cell markers such as ALDH1A1, CD44, SOX2, and Nanog and β-actin in cadmium exposed MTF1 over expressed BPH1 cells. **B**. Spheroid assay for cadmium exposed MTF1 over expressed BPH1 cells. **C**. Immunoblot of stem cell markers such as ALDH1A1, CD44, SOX2, and Nanog and β-actin in cadmium exposed ZIC2 over expressed BPH1 cells. **D**. Immunoblot of EMT markers such as E-cadherin, β-catenin, Slug, and β-actin in cadmium exposed MTF1 overexpressed BPH1 cells. **E.** Invasion assay for cadmium exposed MTF1 overexpressed BPH1 cells.** F.** Immunoblot of EMT markers such as E-Cadherin, β-catenin, Slug, and β-actin in cadmium exposed ZIC2 overexpressed BPH1 cells.

Next, we examined the impact of MTF1 and ZIC2 overexpression on the survival of BPH1 cells. Our observations indicated that overexpression of these two transcription factors reduced the levels of cleaved PARP while increasing the expression of survival markers such as Bcl2, Bcl-xL, p65, and XIAP (Fig- 5C&D). We also noted changes in stemness and EMT in CTBPH1 cells following the silencing of MTF1 and ZIC2. To investigate the potential of enhancing stemness and EMT in BPH1 cells, we over-expressed MTF1 and ZIC2 and found that over-expression elevated the levels of stem cell markers such as Nanog, ALDH1A1, SOX2 and CD44 (Fig 6A &C). We further observed that MTF1 overexpression enhanced the sphere-forming ability of BPH1 cells (Fig 6B).

Furthermore, MTF1 and ZIC2 overexpression upregulated mesenchymal markers, including β-catenin, and Slug. (Fig 6D & F). Interestingly, exposure to cadmium also enhanced the expression of both stem cell and mesenchymal markers. Additionally, MTF1 overexpression promoted invasion of BPH1 cells (Fig 6E). These findings suggest that overexpression of MTF1 and ZIC2 in BPH1 cells mimic the effect of cadmium exposure, indicating that these signaling pathways play a critical role in regulating cellular processes such as stemness, survival, and EMT.

### The silencing of MTF1 and ZIC2 significantly hinders tumor growth in nude mice

**Figure 7:**
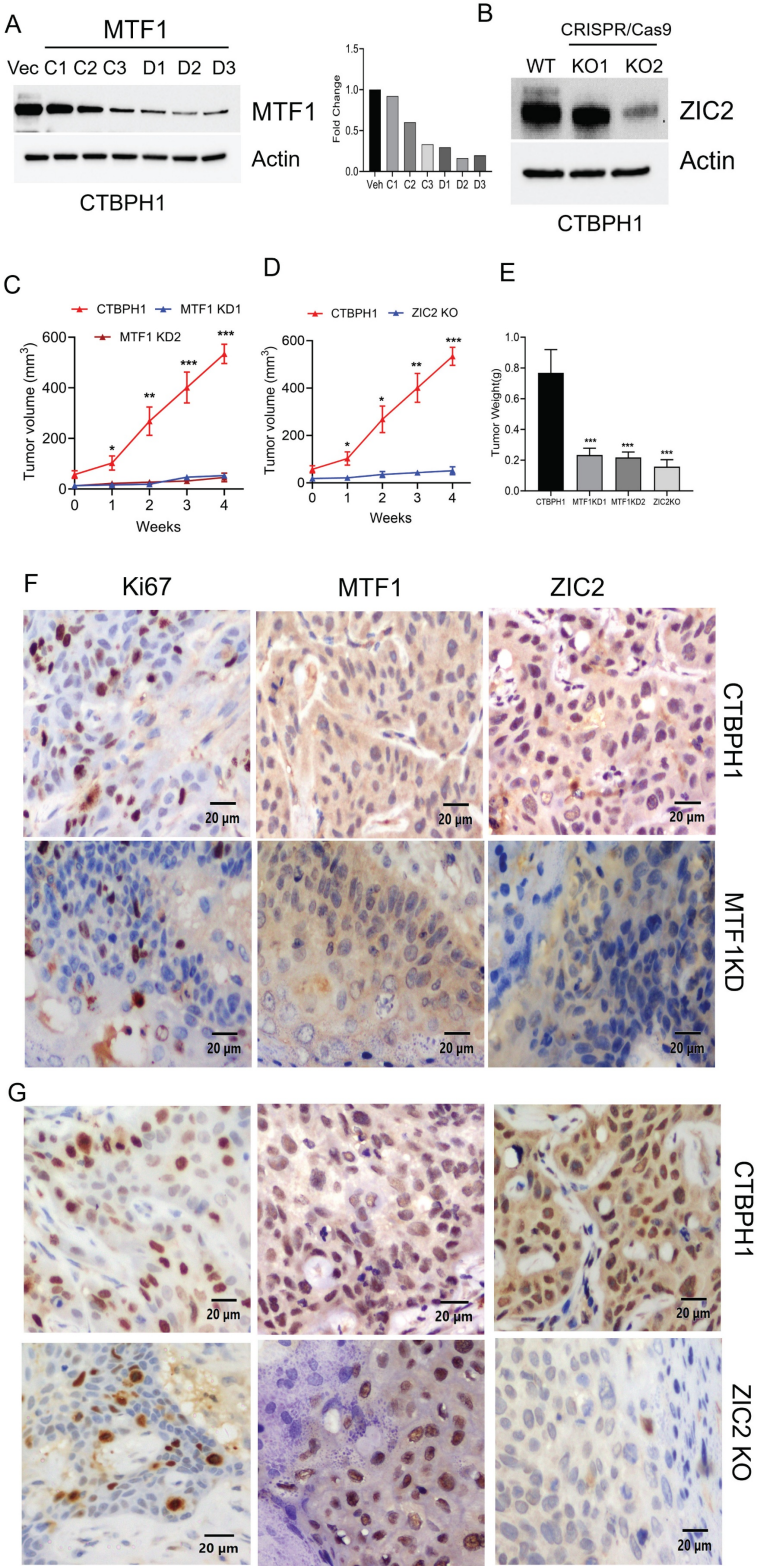
**The silencing of MTF1 and ZIC2 significantly hampers tumor growth in nude mice. A&B**. Immunoblot of MTF1, ZIC2, and β-actin in MTF1 stable knockdown CTBPH1 cells. **C&D**. Tumor growth of CTBPH1 control, MTF1 shRNA knockdown, and ZIC2 knockout CTBPH1 cells subcutaneously implanted in the flanks of nude mice measured twice weekly (n=6). **E**. Tumor weight of CTBPH1, MTF1 knockdown, and ZIC2 knockout tumor. **F&G**. immunohistochemical analysis of tissue section derived from MTF1 ShRNA knockdown, ZIC2 knockout, and CTBPH1 tumors for MTF1, ZIC2, and Ki67 expression.

Our prior research demonstrated that CTBPH1 cells exhibit a malignant phenotype when xenotransplanted into mice 27. To investigate whether silencing MTF1 and knocking out ZIC2 could reduce the tumorigenic potential of CTBPH1 cells, we utilized MTF1 shRNA for silencing MTF1 and generating a CTBPH1 cells with stable knockdown of MTF1 and employed CRISPR-Cas9 to knock out ZIC2 in CTBPH1 cells. The expression levels of MTF1 and ZIC2 were confirmed through western blot analysis (Fig. 7A & B). This process resulted in the establishment of six distinct MTF1 knockdown variants (MTF1C1-C3, MTF1D1-D3) and two CRISPR/cas9 ZIC2 knock out variants (ZIC2KO1 and ZIC2KO2) of CTBPH1 cells. Next, we subcutaneously injected these CTBPH1 cells (which had stable knockdown of MTF1 and ZIC2) into NU/J mice. The control vector-transfected CTBPH1 cells exhibited aggressive growth, while the MTF1 knockdown and ZIC2 CRISPR/cas9 knockout cells significantly inhibited tumor growth and reduced tumor weight (Fig. 7C - E). Tumors derived from MTF1 knockdown and ZIC2 CRISPR/cas9 knockout CTBPH1 cells showed a marked decrease in the expression of the proliferation marker Ki67. Additionally, we observed reduced expression levels of both ZIC2 and MTF1 in MTF1-knockdown CTBPH1 tumors, whereas MTF1 expression remained relatively unchanged in ZIC2 knockout tumors (Fig. 7F and G). This finding further validates our *in vitro* results.

**Figure 8:**
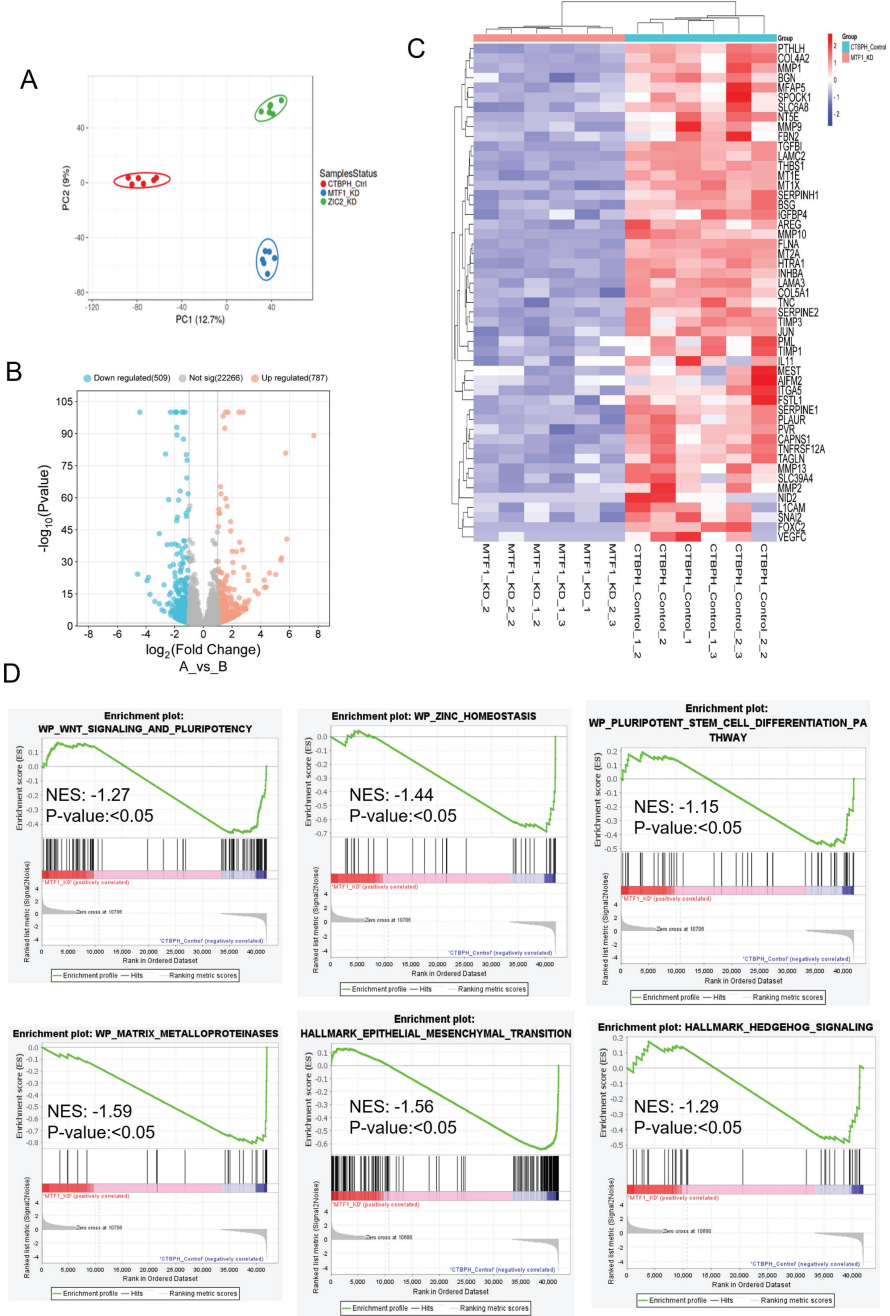
** mRNA seq analysis of MTF1shRNA knockdown and CTBPH1 tumors. A**. PCA plot showing MTF1_KD and ZIC2_KO CTBPH1 xenograft tumors against control examined through RNA-seq gene expression data. The data is colored according to condition (CTBPH1_control, MTF1_KD, and ZIC2_KO). **B**. The volcano plot analysis of differentially expressed genes between MTF1shRNA knockdown against shRNA control is plotted on the X axis, and false discovery rate (FDR) significance is plotted on the Y axis (-log10 scale). The grey dots represent no significant change, the red dots represent logFC of >1 and FDR < 0.05, and the blue dots represent logFC < -1 and FDR < 0.05. **C&D**. Heat map and gene enrichment analysis showed the crucial genes enriched at the WNT signaling and pluripotency, ZINC homeostasis, stem cell differentiation, apoptosis modulation, EMT, and hedgehog signaling pathway. Gene set enrichment analysis (GSEA) of MTF1shRNA knockdown tumor compared to shRNA control tumors

We assessed the transcriptional effects of MTF1 and ZIC2 inhibition using RNA sequencing in CTBPH1 xenograft tumors. Principal component analysis revealed significant differences among CTBPH1 control, MTF1 knockdown, and ZIC2 CRISPR/cas9 knockout tumors (Fig. 8A). To identify changes in gene expression across these tumors, we performed a volcano plot-based analysis on MTF1 knockdown, ZIC2 CRISPR/cas9 knockout, and CTBPH1 control tumors. mRNA sequencing revealed that 787 genes were significantly upregulated (logFC ≥ 1, p-value < 0.05) and 509 genes were significantly downregulated (logFC ≤ -1, p-value ≤ 0.05) in MTF1 knockdown tumors compared to vehicle-treated CTBPH1 tumors (Fig. 8B). Importantly, MTF1 knockdown decreased the expression of genes such as *MMP1, MT1X, TIMP3, JUN, MMP2, FOXC2*, and *VEFGFC*, among others (Fig. 8C). Geneset enrichment analysis showed a reduction in Hedgehog signaling (NES: -1.29) (p<0.05), epithelial-mesenchymal transition (EMT) (NES: -1.56) (p<0.05), WNT signaling, pluripotency (NES: -1.27) (p<0.05), the pluripotent stem cell pathway (NES: -1.15) (p<0.05), the MMP pathway (NES: -1.59) (p<0.05), and the ZINC homeostasis pathway (NES: -1.44) (p<0.05) following MTF1 knockdown (Fig. 8D). These findings support the idea that MTF1 enhances stemness-like traits and facilitates the transformation of BPH1 cells.

**Figure 9:**
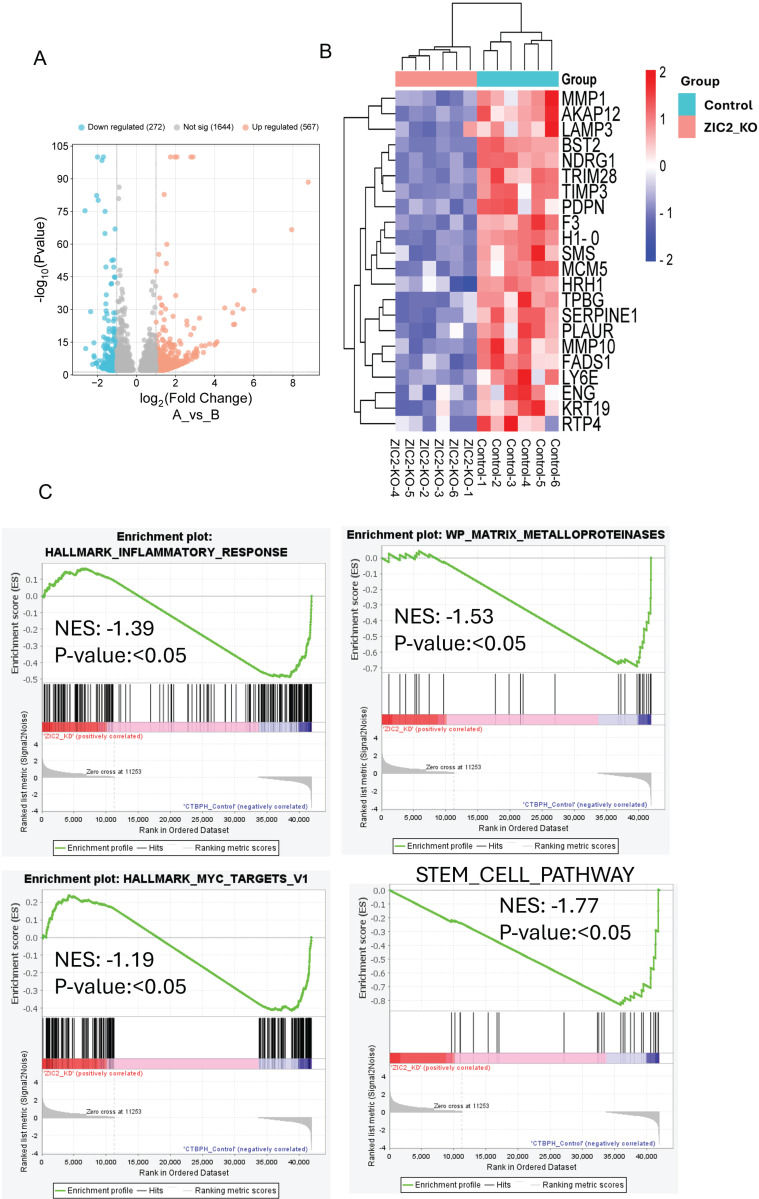
**mRNA seq analysis of ZIC2 CRISPR/Cas9 knockout and CTBPH1 tumors. A**. The volcano plot analysis of differentially expressed genes between ZIC2 knockout against CTBPH1 is plotted on the X axis, and false discovery rate (FDR) significance is plotted on the Y axis (-log10 scale). The grey dots represent no significant change, the red dots represent logFC of >1 and FDR < 0.05, and the blue dots represent logFC < -1 and FDR < 0.05. **B&C**. Heat map and Gene enrichment analysis showed the crucial genes enriched at the inflammatory response, myc targets, matrix metalloproteinases, and stem cell pathway. Gene set enrichment analysis (GSEA) of ZIC2 knockout tumor compared to control tumors.

Similarly, we found that 567 genes were upregulated (logFC ≥ 1, p-value < 0.05) and 272 genes were downregulated (logFC ≤ -1, p-value ≤ 0.05) in ZIC2 CRISPR/cas9 knockout tumors compared to CTBPH1 tumors treated with a vehicle (Fig. 9A). Notably, ZIC2 CRISPR/cas9 knockout led to the downregulation of several genes associated with pathways such as *MMP1, LAMP3, TIMP3, MMP10, KRT19, and SERPINE* (Fig. 9B). We subsequently analyzed the functional enrichment of pathways associated with ZIC2 knockout, indicating a downregulated in the inflammatory pathway (NES: 1.39) (p<0.05), the MMP pathway (NES: -1.53) (p<0.05), the stem cell pathway (NES: 1.77) (p<0.05), and the Myc pathway (NES: -1.19) (p<0.05) (Fig. 9C).

Overall, our *in vivo* studies demonstrate that the MTF1 and ZIC2 signaling axis is important for the regulation of proliferation, stemness, and EMT. All of this is necessary for cadmium to convert benign prostate epithelial cells into tumors.

## Discussion

Cadmium, a common occupational and environmental contaminant, has been associated with human prostate cancer 30, 31. The primary route of cadmium exposure in the general population is through inhalation, primarily from cigarette smoking 32. A single pack of cigarettes contains approximately 1 to 3 micrograms of cadmium, leading to urinary cadmium levels that are twice as high in smokers compared to non-smokers 33. Understanding the environmental and molecular factors that influence the progression of prostate cancer is of significant translational importance. While several studies have highlighted the link between cadmium exposure and prostate cancer progression, the specific cellular mechanisms by which cadmium induces the transformation of BPH1 cells remain largely unclear. Our research provides insights into cadmium-induced carcinogenesis mechanisms, establishing a foundation for future studies and potential therapeutic strategies 34-36.

We previously demonstrated that ZIC2 and GLI1 are critical mediators of cadmium-induced transformation in BPH1 cells 27. However, the underlying mechanism remains unclear. In this study, we identified MTF1 as a key regulator of cadmium-induced transformation in BPH1 cells, playing a significant role in cell survival and regulating the ZIC2 transcription factor. Our transcriptomic analysis showed that the knockdown of MTF1 alters multiple pathways associated with EMT, oncogenesis, zinc homeostasis, and stem cell differentiation pathways. Additionally, the ZIC2KO tumor transcriptomic data modified pathways related to stem cells, myc, inflammatory response, and matrix metalloproteinases.

These findings significantly enhance our understanding of the molecular mechanisms behind cadmium-induced carcinogenesis and potential therapeutic targets. Moreover, our results reveal that the overexpression of MTF1 and ZIC2 mimics to cadmium exposure inhibits PARP activation while promoting the expression of other survival markers, enhancing stem cell-like characteristics, and facilitating EMT phenotypes in BPH1 cells. This knowledge is crucial for further research and potential therapeutic interventions. MTF1 knockdown in cadmium-exposed CTBPH1 cells modulates these pathways, underscoring MTF1's pivotal role in mediating the effects of cadmium. Our data strongly suggest that chronic exposure to cadmium induces MTF1 expression, which drives the transformation of BPH1 cells into malignant phenotypes.

The MTF1 is a six-zinc finger protein that plays a crucial role in activating the production of metallothionein in response to heavy metals, such as zinc and cadmium 37. In the context of cadmium-induced cellular transformation, MTF1 is essential for regulating the expression of pro-survival genes, promoting transformed cells' survival. This function of MTF1 is particularly significant for understanding the molecular mechanisms underlying cadmium-induced carcinogenesis. We observed that the knockout of MTF1 is lethal to both BPH1 and CTBPH1 cells. It appears the cells require an endogenous level of MTF1 expression for optimal functioning. Previous studies have shown that complete knockout of MTF1 in a mouse model result in an embryonic lethal phenotype characterized by liver degradation; furthermore, hepatocytes from MTF1 null mutants were lost within just a few days of cultivation 38. Importantly, the phenotypic changes we aimed to monitor in the absence of MTF1 could be achieved through MTF1 ShRNA knockdown. Consequently, we employed stable MTF1 knockdown in our cellular assays and for generating MTF1 knockdown cells for xenograft studies.

Cadmium activates MTF1, a transcription factor that binds to DNA and increases the expression of metal detoxification genes, mainly metallothioneins. This facilitates the sequestration of cadmium in the cells. Moderate cadmium exposure induces MTF1 activation, whereas increased amounts of cadmium impair this pathway and can lead to cell death 39. Moreover, MTF1 operates within a complex regulatory network that includes other factors like ZIC2 and GLI1. It binds to the metal regulatory element (MRE) in the promoters of target genes, thereby regulating their expression. The activation of MTF1 is mediated by various molecular mechanisms that help maintain metal and redox homeostasis. In response to heavy metals and oxidative stress, MTF1 triggers the transcription of metal-responsive genes 11, 40.

Notably, overexpression of MTF1 has been observed in human intrahepatic cholangiocarcinoma, where it contributes to tumor differentiation, vascular invasion, and poor prognosis 41. Additionally, MTF1 overexpression plays a significant role in the progression of various cancers, including prostate cancer 42. Loss of MTF-1 expression has inhibited tumor development by increasing matrix collagen deposition and decreasing vascular density 43. To investigate the role of MTF1, we performed functional assays in MTF1 knockdown CTBPH1 and control cells. Our findings revealed that the MTF1 knockdown significantly reduced cell proliferation, sphere formation, migration, and invasion in CTBPH1 cells. Additionally, silencing MTF1 through either a pharmacological inhibitor or shRNA in the presence of cadmium led to a downregulation of survival, stem cell, and EMT markers. Interestingly, cadmium failed to induce the expression of oncogenic markers in MTF1-silenced cells, confirming that activation of stemness and tumorigenic pathways was dependent on MTF1 expression. Our findings uncover a novel mechanism by which MTF1 promotes cadmium-induced tumorigenesis in CTBPH1 cells, opening up new avenues for research in this field.

ZIC2, a member of the *ZIC2* gene family, has demonstrated carcinogenic properties 44, 45. The *ZIC2* gene encodes the zinc finger protein ZIC2, which interacts with DNA and proteins 46, 47. Numerous studies have reported abnormal expression of ZIC2 in various solid tumors, including breast cancer 48, colon cancer 49 and renal clear cell carcinoma 50. Previous studies suggest that ZIC2 can serve as a prognostic biomarker, correlating with poor prognosis and high immune infiltration of liver cancer 51. Additionally, ZIC2 has been shown to play a significant role at the molecular and cellular levels in the formation and progression of prostate carcinoma 17. Our study demonstrates that overexpressing ZIC2 in BPH1 cells exposed to cadmium increased the expression of stem cell markers (ALDH1A1, CD44, and Nanog), which accelerated the EMT process.

Under resting conditions, MTF1 predominantly exists in the cytoplasm; however, it translocates to the nucleus following exposure to metals like Zinc or cadmium 52, 53. The localization of transcription factors within the nucleus plays a crucial role in regulating gene expression during cell differentiation or changes in metabolic state. While most transcription factors are localized in the nucleus 54, others predominantly exist in the cytoplasm and translocate to the nucleus in response to stimuli. A previous study showed that cadmium exposure at toxic concentrations rapidly induces the complete nuclear translocation and activation of the DNA-binding activity of MTF-1. 52, 55. Our study examined acute and chronic cadmium exposure, which led to the accumulation of nuclear MTF1 and ZIC2 expression in CTBPH1 and BPH1 cells.

Furthermore, we found that MTF1 binding sites in the ZIC2 promotor. Notably, a cadmium-induced increase in MTF1 binding to the ZIC2 promoter suggests a possible mutual exclusivity between these transcription factors in regulating MTF1 transcription. This mutual exclusivity may have significant implications for understanding the regulatory network of MTF1 and ZIC2 in the context of cadmium-induced transformation. Thus, cadmium exposure may facilitate the cytoplasmic MTF1 and ZIC2 translocation into the nucleus, where they collaborate to activate transcription. Our findings demonstrate that acute and chronic cadmium exposure promotes nuclear translocation of MTF1, thereby activating its downstream targets.

Interestingly, our immunoprecipitation data (Fig 1F) revealed that MTF1 and ZIC2 interact with each other and jointly drive tumorigenesis and progression of cadmium-transformed BPH1 cells. Metal regulatory elements (MREs) are specific DNA sequences that facilitate the activation of genes in response to heavy metals. These elements are typically located in the promoter regions of genes that respond to such metals 56. MRE-binding transcription factor-1 (MTF-1) plays a crucial role by interacting with MREs to initiate gene transcription 57. MREs specifically mediate the transcriptional response of metallothionein genes to cadmium exposure 58. To investigate whether cadmium exposure contributes to transcriptional changes in CTBPH1 cells, we overexpressed a luciferase MRE promotor in CTBPH1 cells and then treated the cells with cadmium. Our findings revealed that cadmium exposure significantly increased MRE luciferase activity, indicating that cadmium enhances MRE transcriptional activity.

Our ChIP studies confirmed that MTF1 is stably associated with the ZIC2 promoter in a nucleoprotein environment. Further investigation of the transcriptional regulation of the ZIC2 gene by MTF1 revealed potential MTF1 binding sites on the ZIC2 promotor, which we validated in cell culture through luciferase activity analysis. We observed a significant increase in the luciferase activity in the presence of MTF1, which could be attributed to cadmium exposure. We found that ZIC2 transcription activity was increased in CTBPH1 cells following chronic cadmium exposure. These findings highlight MTF1's crucial role in mediating the effects of cadmium by suggesting that the knockdown of MTF1 in CTBPH1 cells affects several pathways, as confirmed by RNA sequencing.

In conclusion, we found that MTF1 is an upstream factor for ZIC2. We also found that inhibition of the MTF1/ZIC2 axis reduced tumor burden. Specifically, our results demonstrate that proliferation of CTBPH1 cells depends on activation of the MTF1/ZIC2 signaling pathway, which drives stem cell formation and initiates epithelial-mesenchymal transition in cadmium-transformed cells. Inhibition of the MTF1/ZIC2 signaling axis arrests stem cell formation, thereby preventing the survival of cadmium-transformed cells and inhibiting tumor growth in xenograft models. The ability of MTF1 to directly bind ZIC2 is crucial for elucidating the molecular mechanism of cadmium-induced BPH1 transformation as it regulates the expression of multiple genes. Understanding the molecular mechanisms of key regulatory elements contributing to cadmium-induced prostate cancer provides new perspectives for the prevention and treatment of prostate cancer.

## Supplementary Material

Supplementary figures and tables.
