# Supplementary material for: Mechanistic role of metal-responsive transcription factor-1 (MTF1) in cadmium-induced prostate carcinogenesis
Source: Int J Biol Sci. 2025 May 27;21(8):3614–30. doi: 10.7150/ijbs.110174 (PMC12160861; doi:10.7150/ijbs.110174)

Figure 1 A

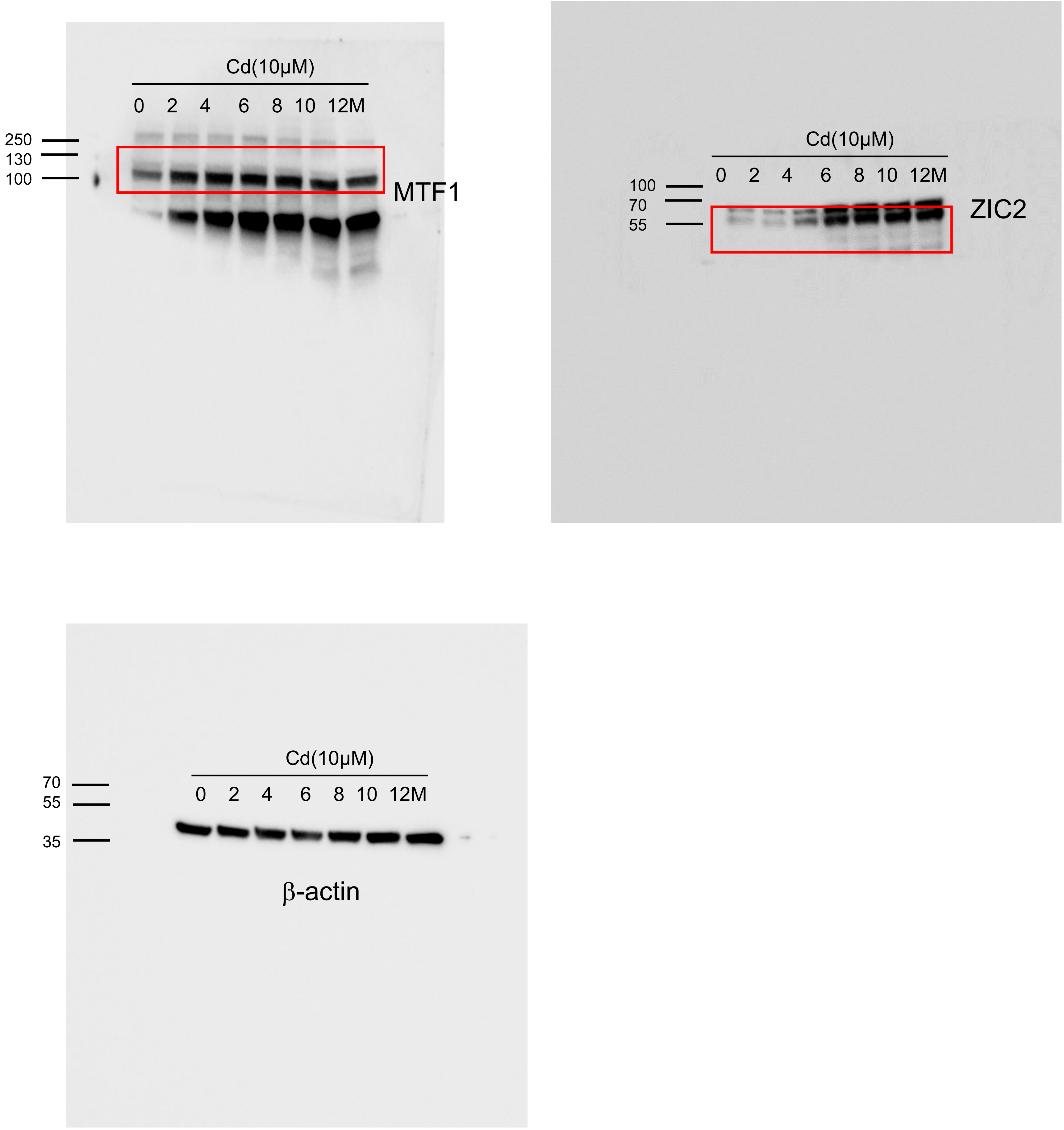
Cd(10μM)
0 2 4 6 8 10 12M
250
130
100
MTF1
Cd(10μM)
0 2 4 6 8 10 12M
100
70
55
ZIC2
Cd(10μM)
0 2 4 6 8 10 12M
70
55
35
β-actin

Figure 1D

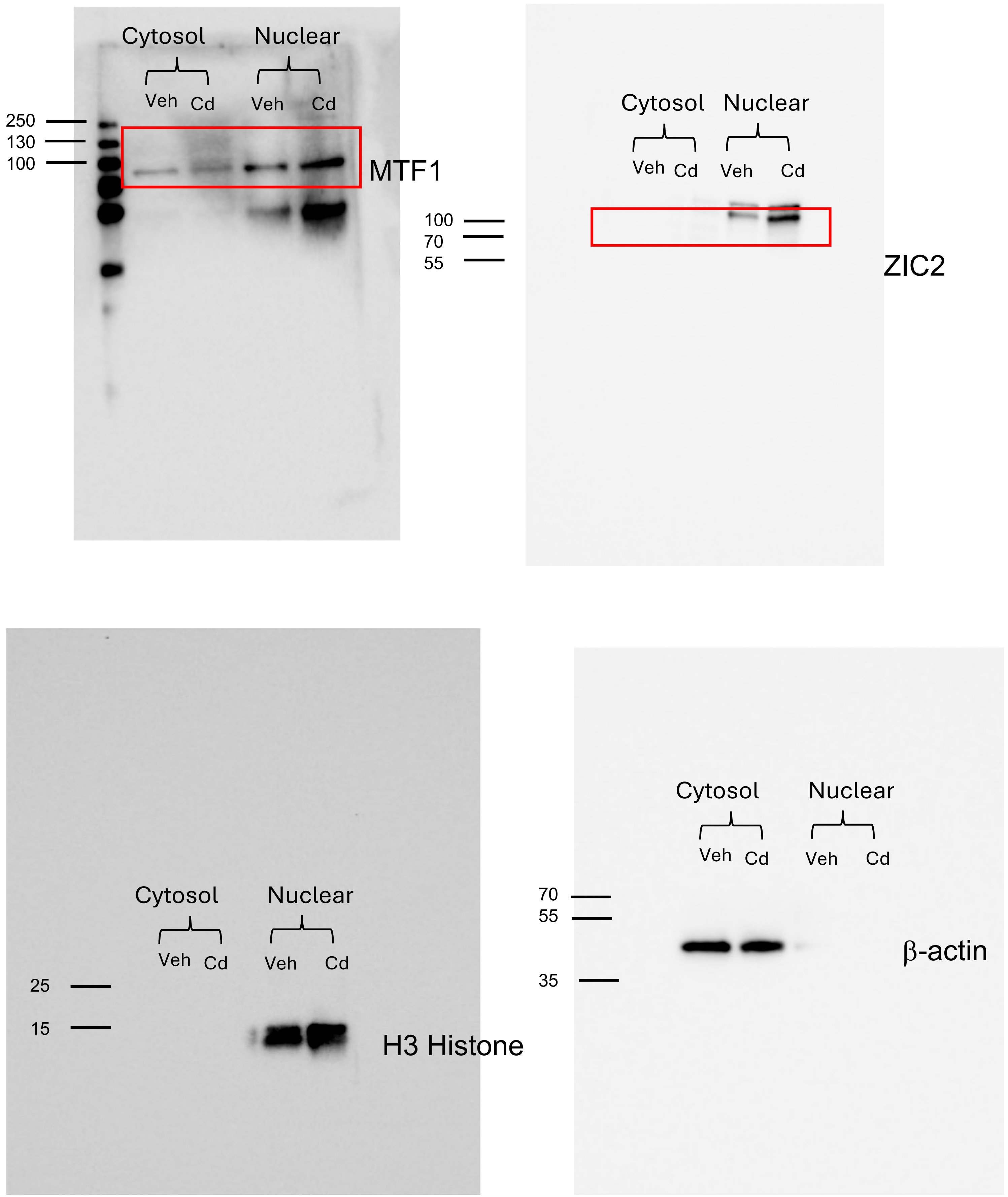
Cytosol
Nuclear
Veh
Cd
Veh
Cd
250
130
100
MTF1
Cytosol
Nuclear
Veh
Cd
Veh
Cd
100
70
55
ZIC2
Cytosol
Nuclear
Veh
Cd
Veh
Cd
25
15
H3 Histone
Cytosol
Nuclear
Veh
Cd
Veh
Cd
70
55
35
β-actin

Figure 1E

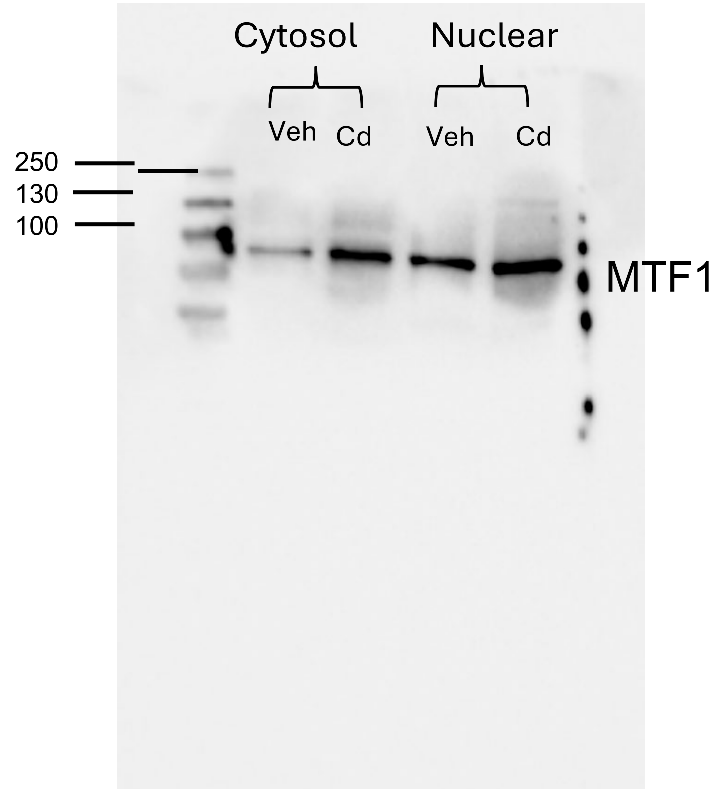
Cytosol
Nuclear
Veh
Cd
Veh
Cd
250
130
100
MTF1

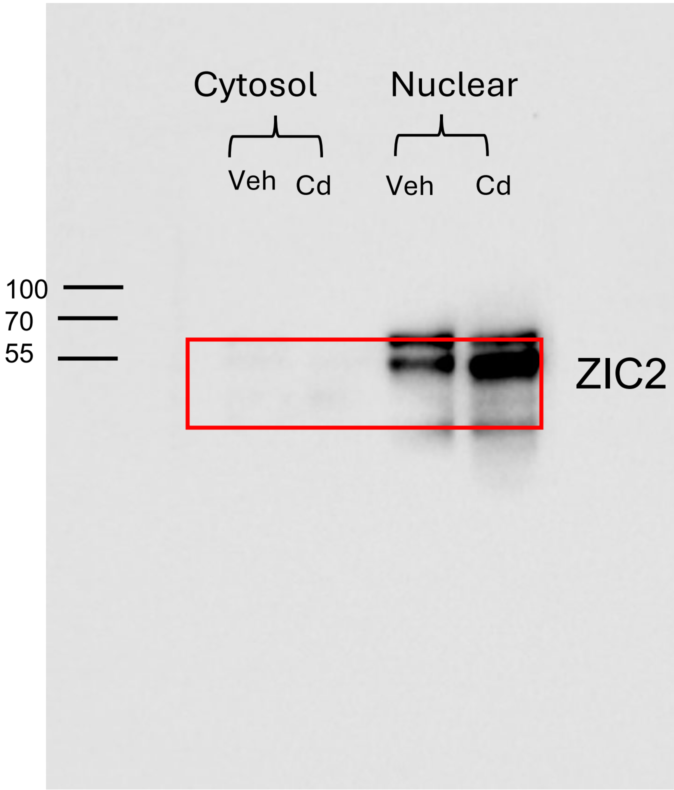
Cytosol
Nuclear
Veh
Cd
Veh
Cd
100
70
55
ZIC2

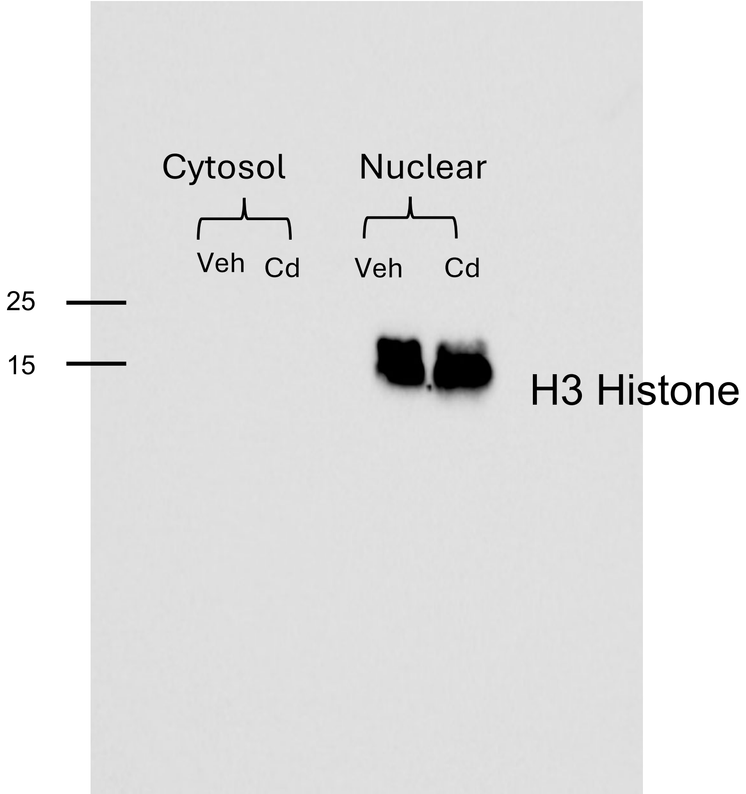
Cytosol
Nuclear
Veh
Cd
Veh
Cd
25
15
H3 Histone

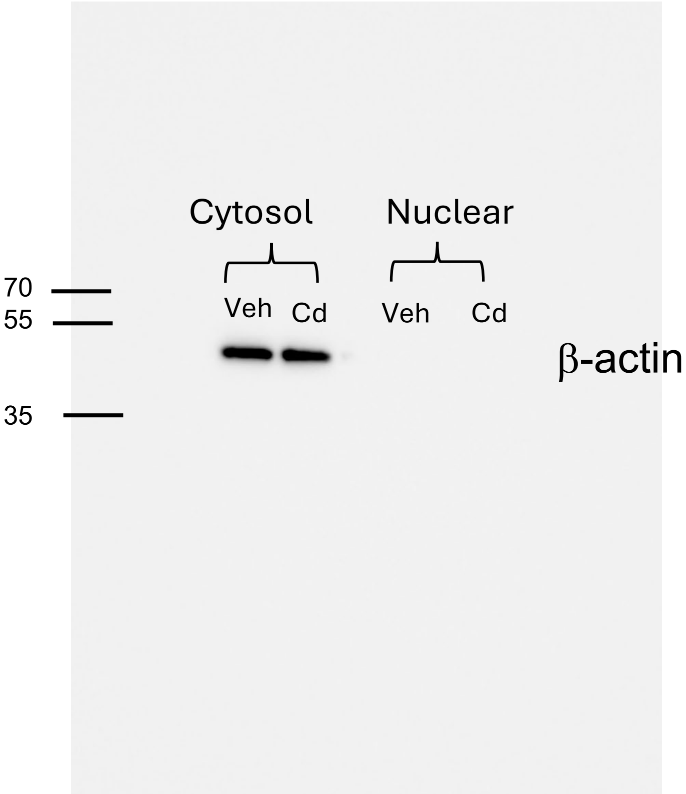
Cytosol
Nuclear
Veh
Cd
Veh
Cd
70
55
35
β-actin

Figure 1F

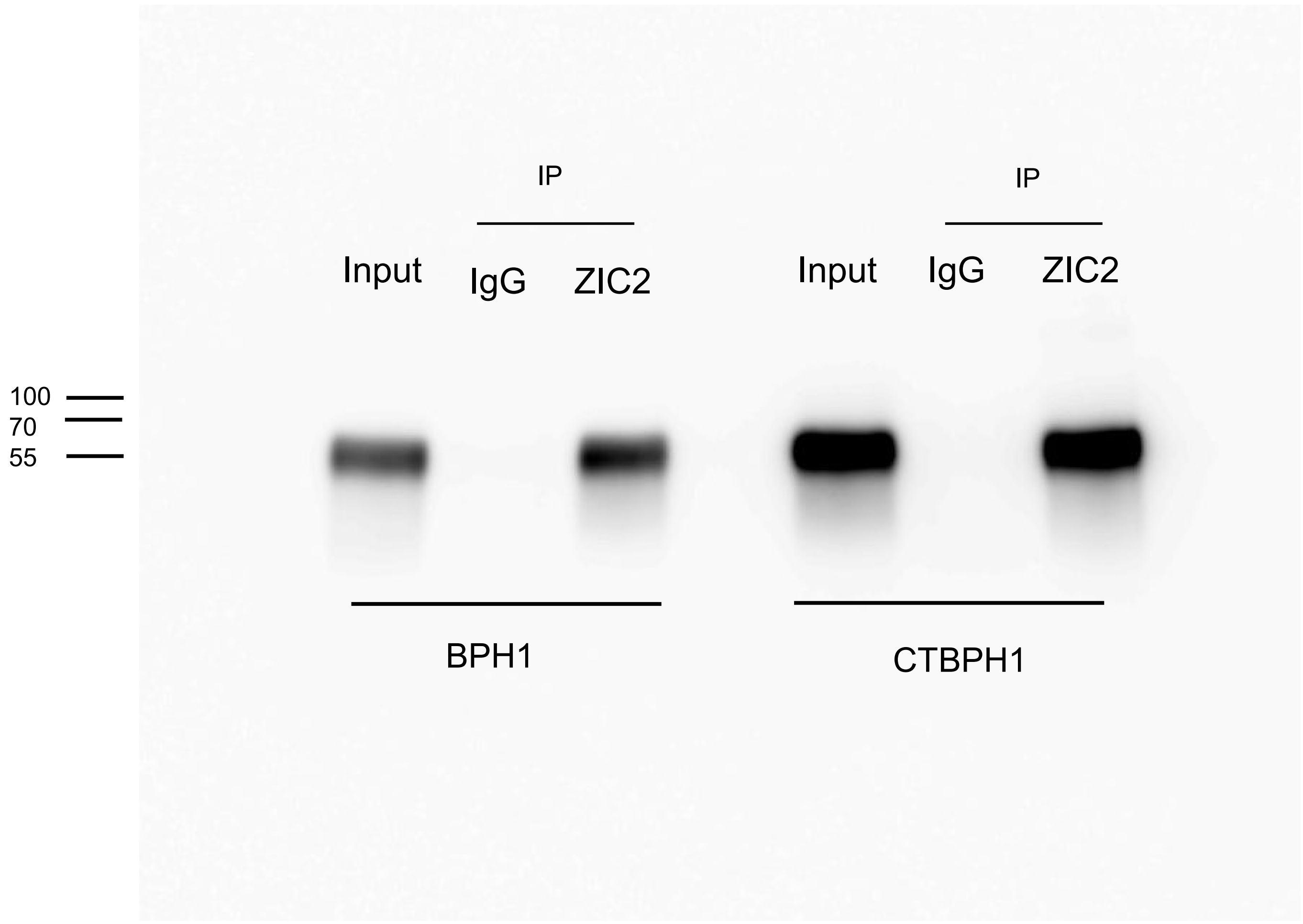

IP:ZIC2

WB:MTF1

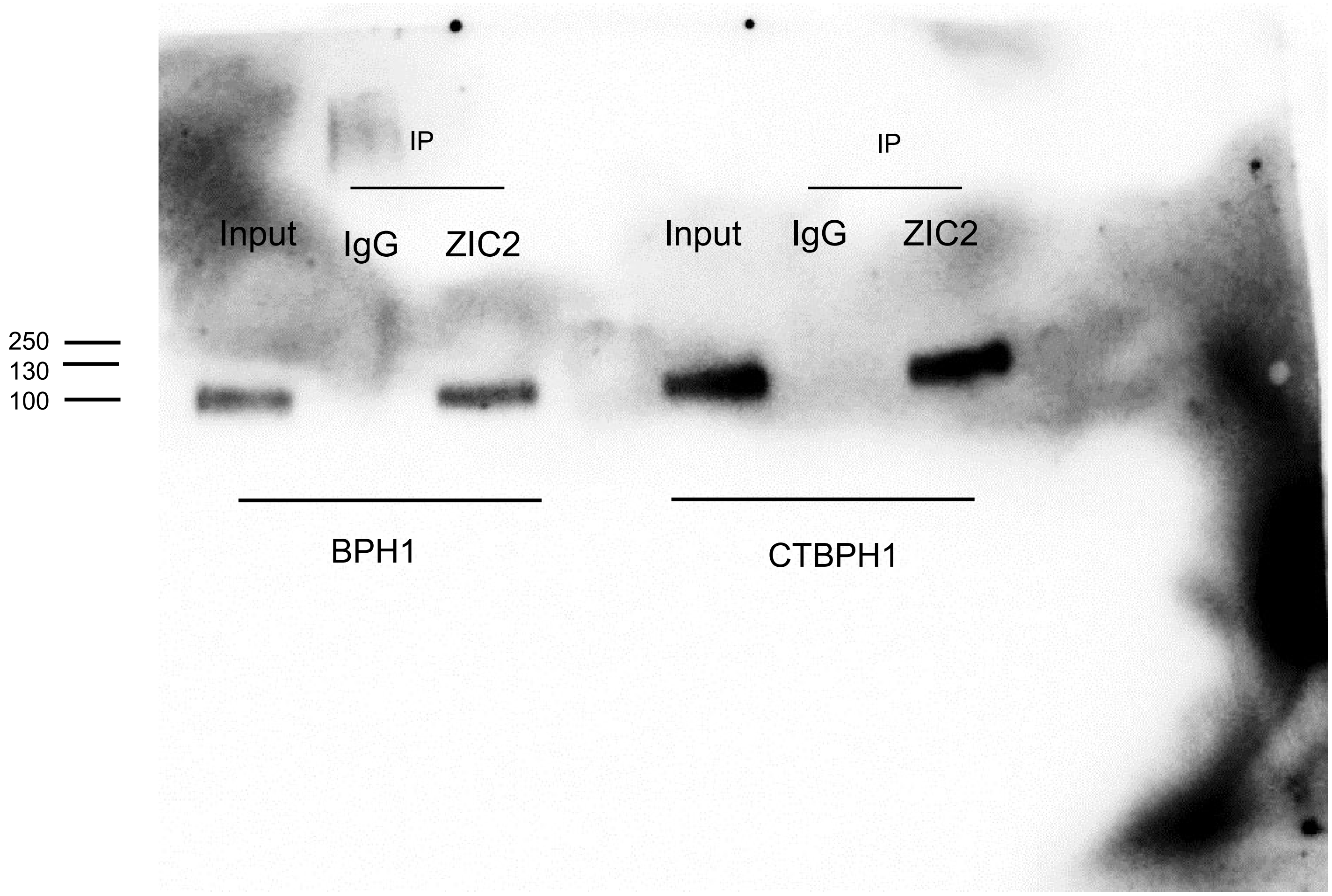

Figure 1K

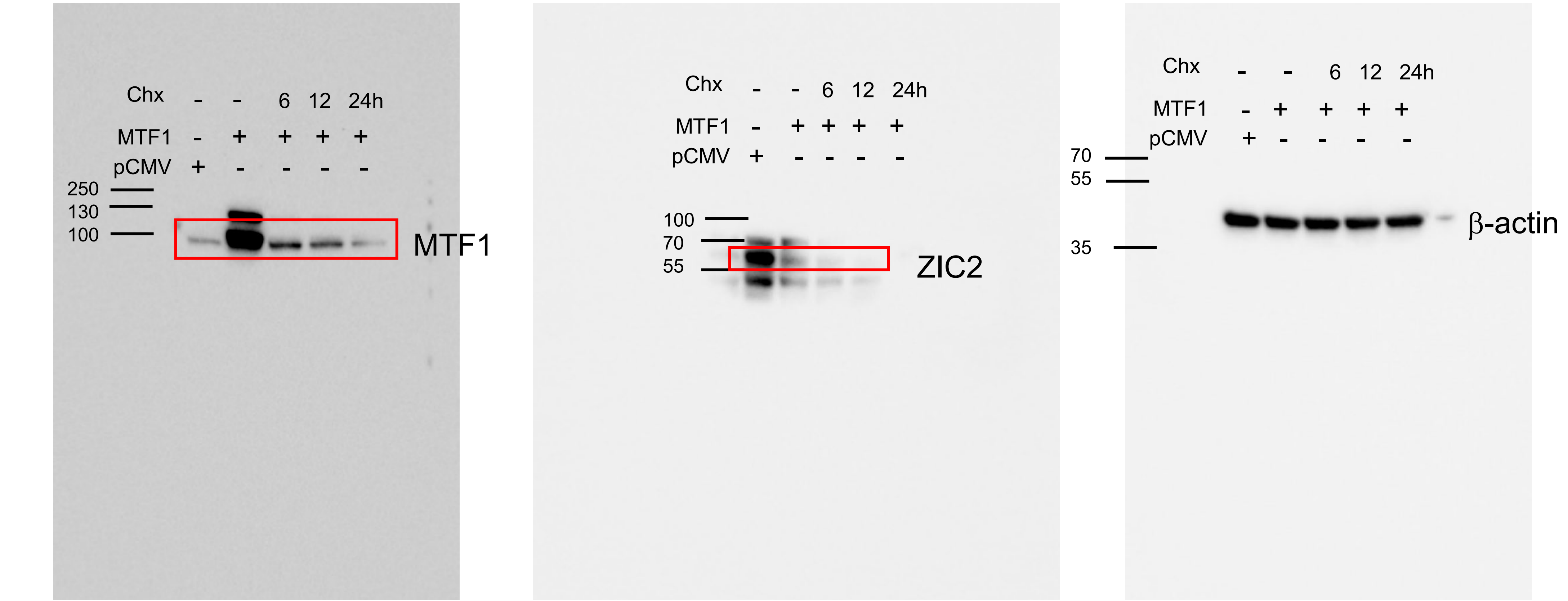

Figure 1L

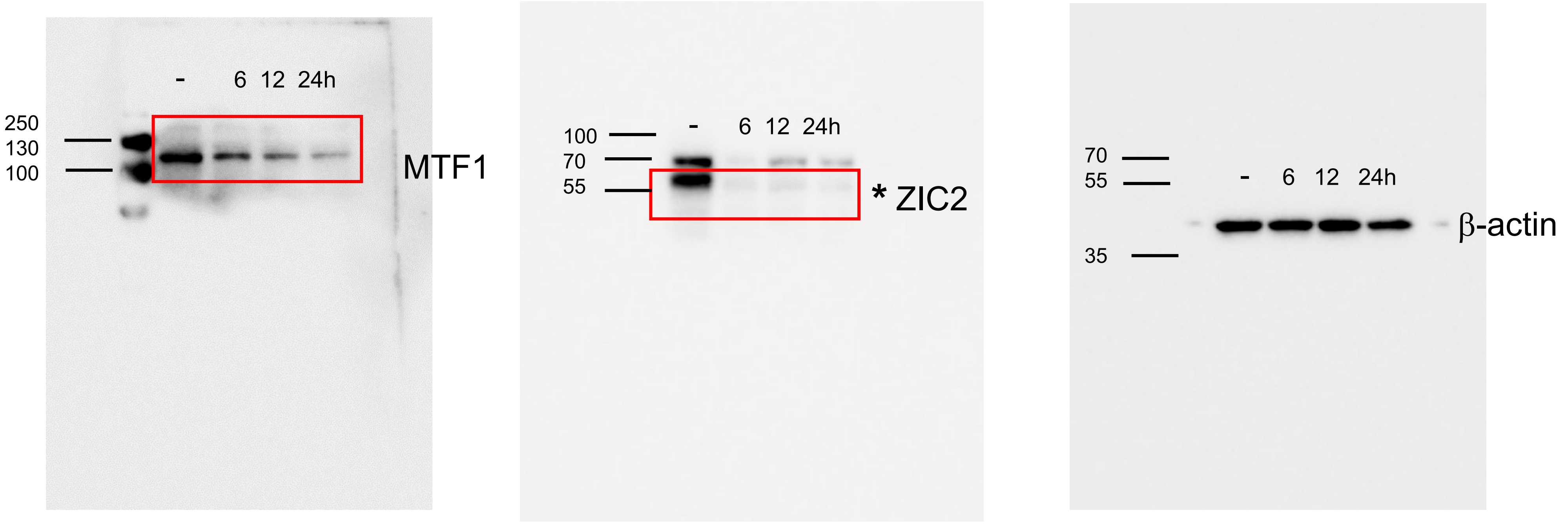

Figure 2A

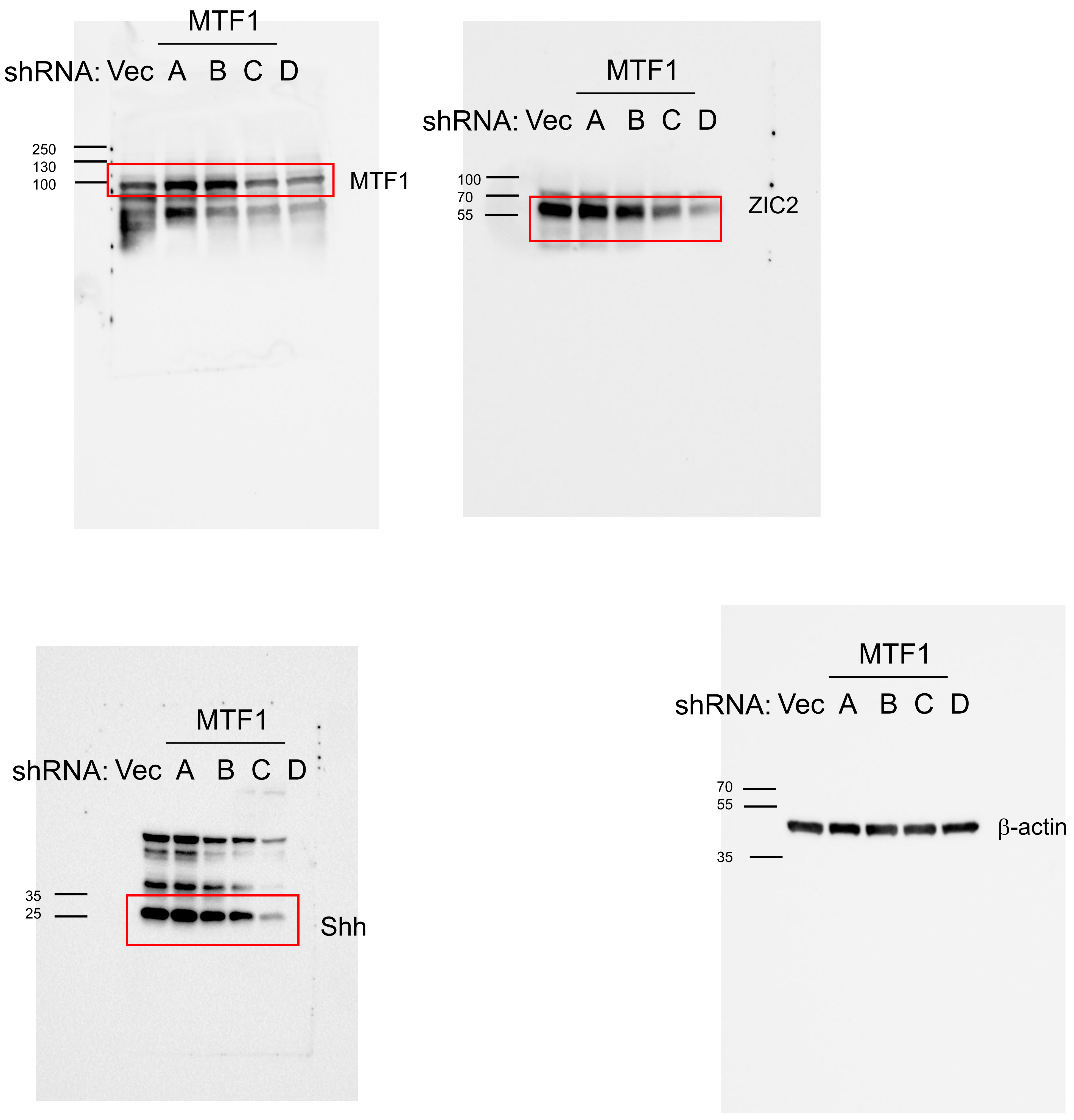
MTF1
shRNA: Vec A B C D
250
130
100
MTF1
MTF1
shRNA: Vec A B C D
100
70
55
ZIC2
MTF1
shRNA: Vec A B C D
35
25
Shh
MTF1
shRNA: Vec A B C D
70
55
35
β-actin

Figure 2B

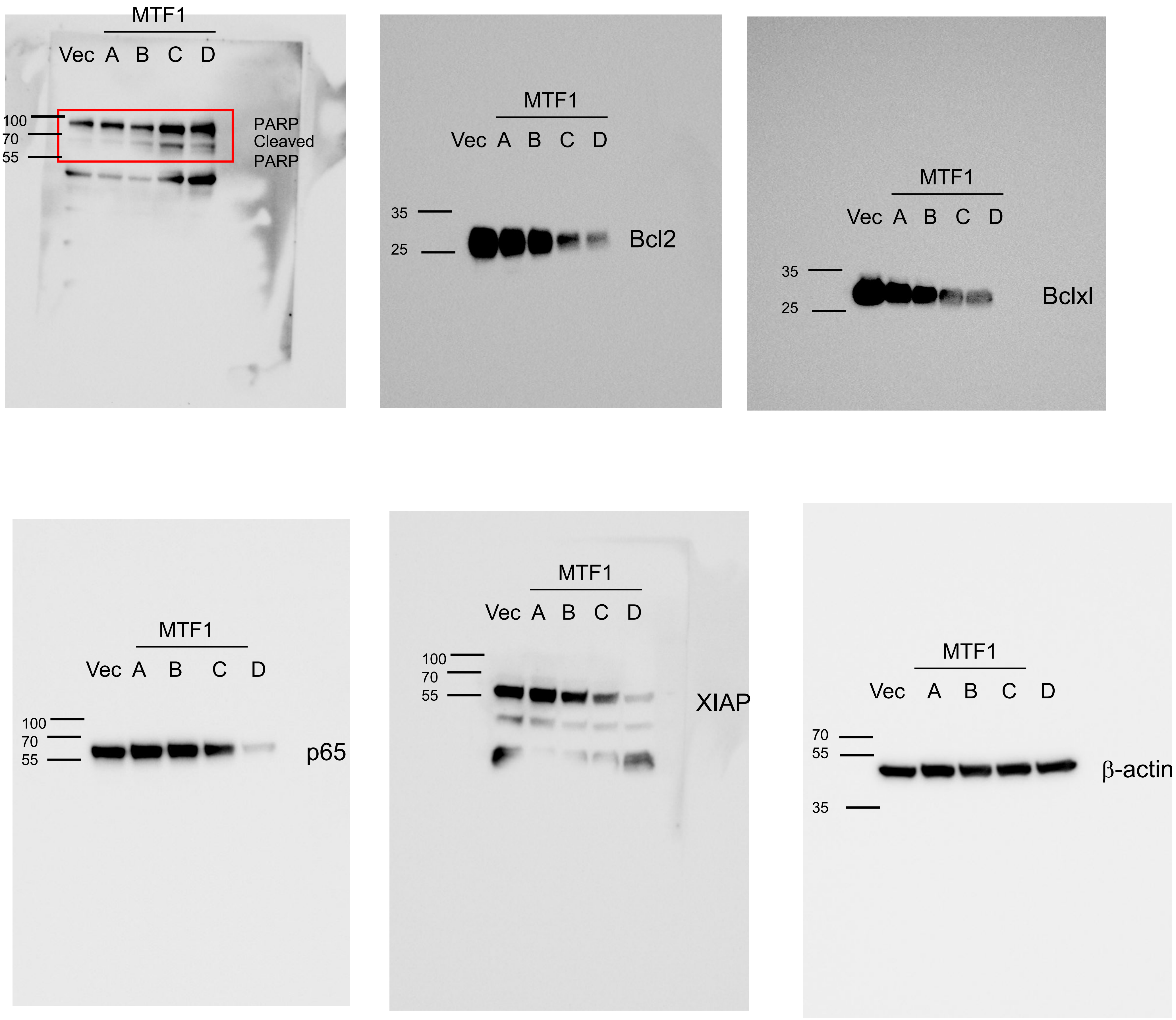
MTF1
Vec A B C D
100
70
55
PARP
Cleaved
PARP
MTF1
Vec A B C D
35
25
Bcl2
MTF1
Vec A B C D
35
25
Bclxl
MTF1
Vec A B C D
100
70
55
p65
MTF1
Vec A B C D
100
70
55
XIAP
MTF1
Vec A B C D
70
55
35
β-actin

Figure 2D

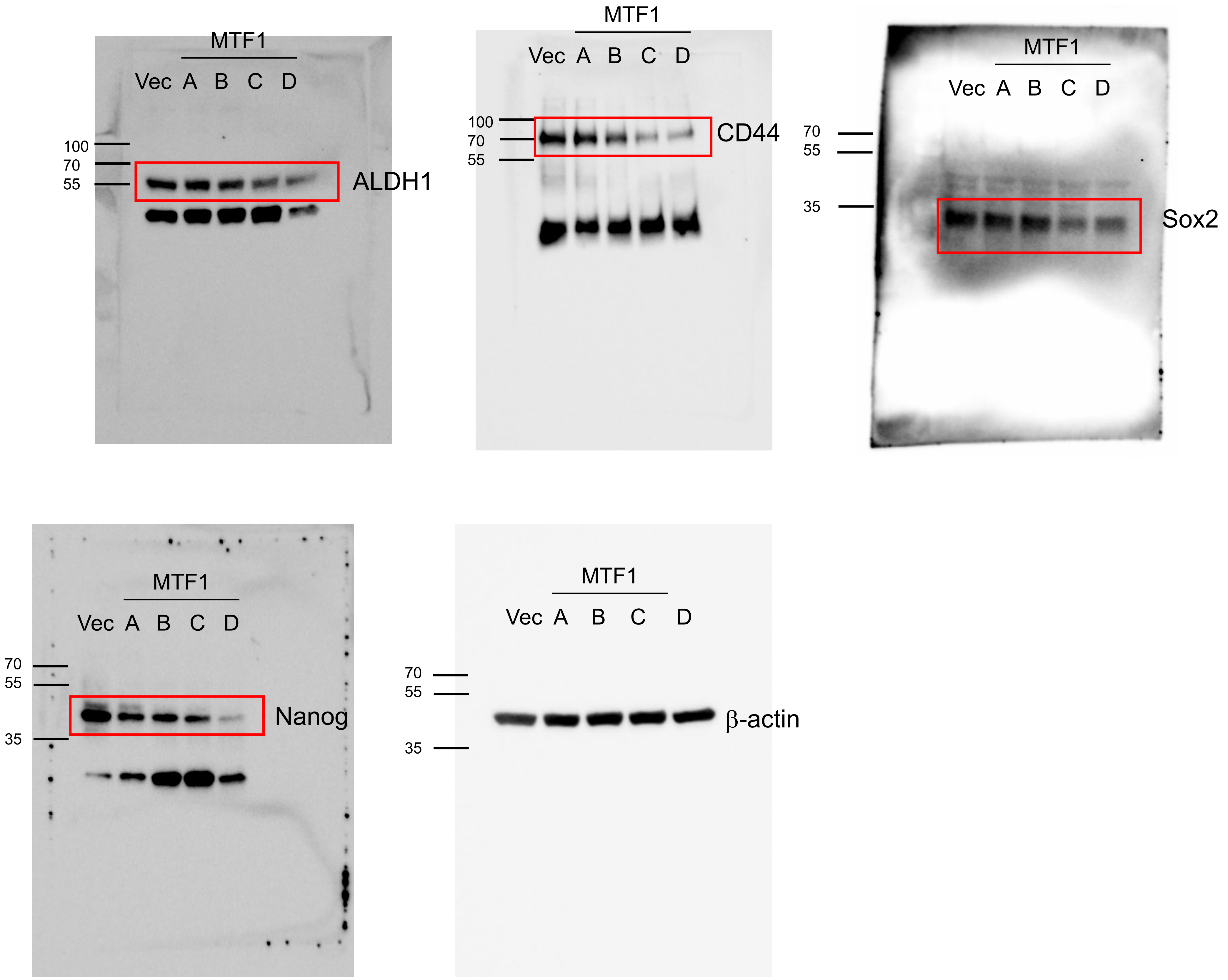
MTF1
Vec A B C D
100
70
55
ALDH1
MTF1
Vec A B C D
100
70
55
CD44
MTF1
Vec A B C D
70
55
35
Sox2
MTF1
Vec A B C D
70
55
35
Nanog
MTF1
Vec A B C D
70
55
35
β-actin

Figure 2E

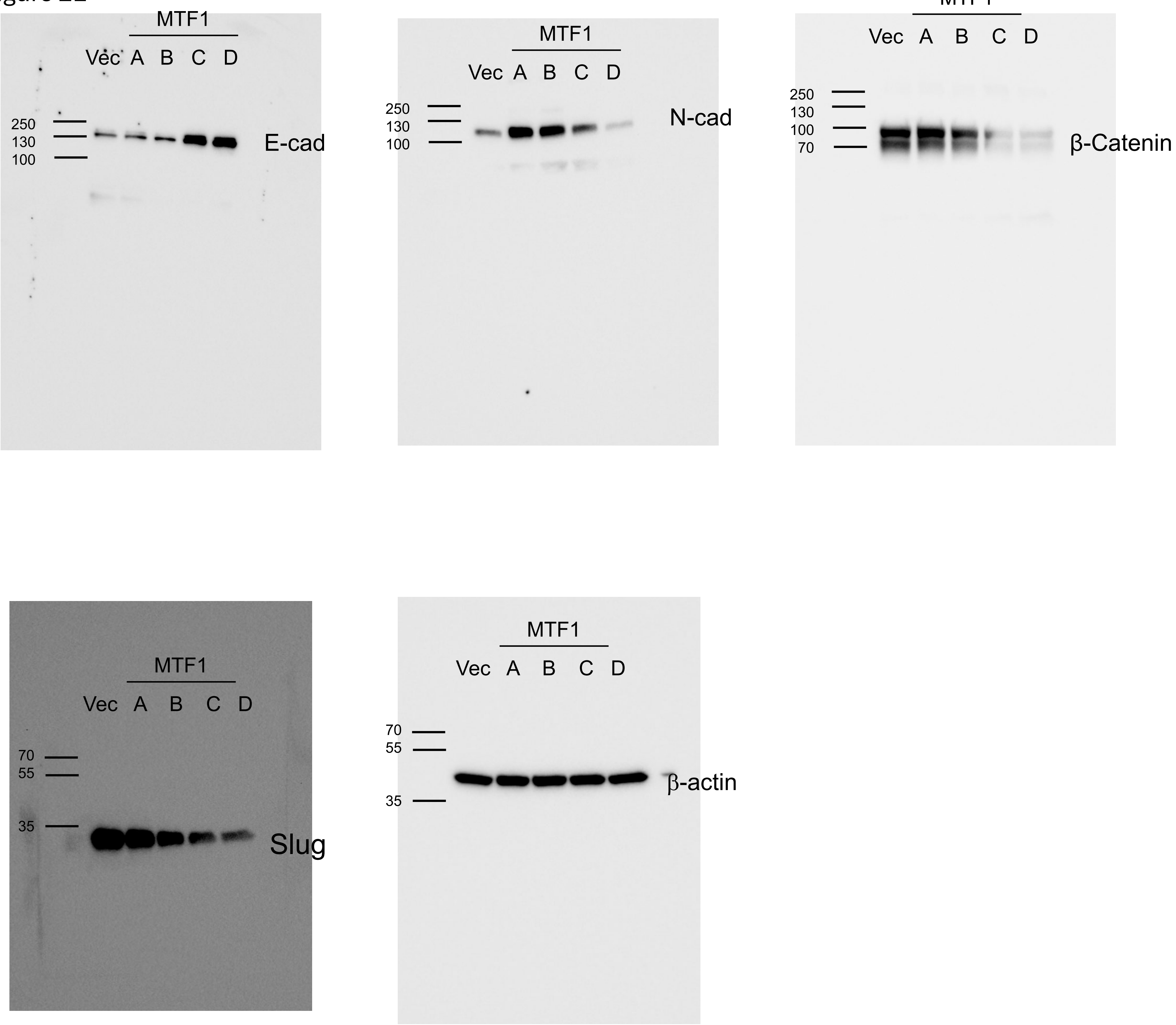
MTF1
Vec A B C D
250
130
100
E-cad
MTF1
Vec A B C D
250
130
100
N-cad
MTF1
Vec A B C D
250
130
100
70
β-Catenin
MTF1
Vec A B C D
70
55
35
Slug
MTF1
Vec A B C D
70
55
35
β-actin

Figure 3A

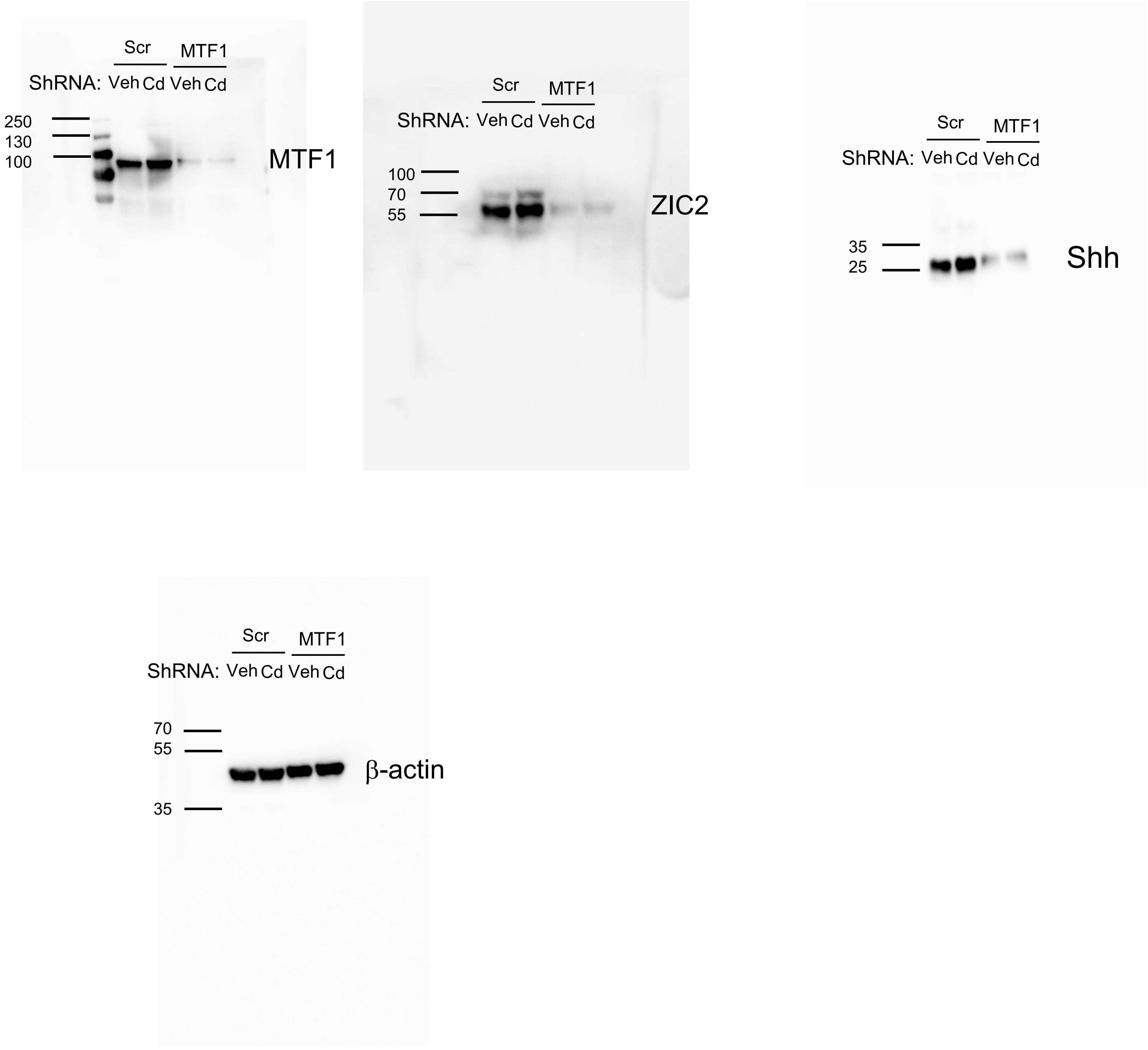

Scr
MTF1
ShRNA: Veh Cd Veh Cd
250
130
100
MTF1
Scr
MTF1
ShRNA: Veh Cd Veh Cd
100
70
55
ZIC2
Scr
MTF1
ShRNA: Veh Cd Veh Cd
35
25
Shh
Scr
MTF1
ShRNA: Veh Cd Veh Cd
70
55
35
β-actin

Figure 3C

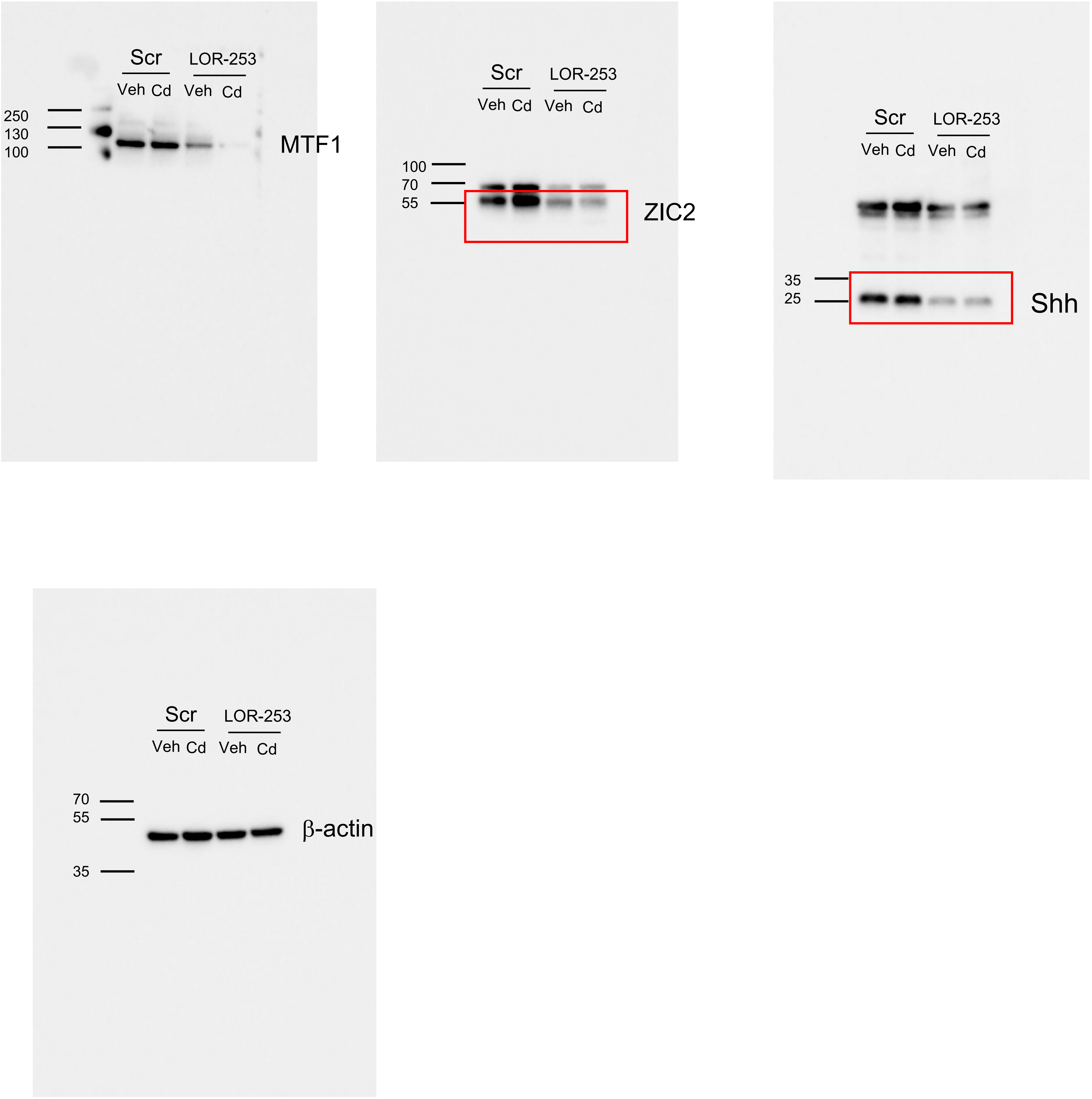
Scr
LOR-253
Veh Cd Veh Cd
250
130
100
MTF1
Scr
LOR-253
Veh Cd Veh Cd
100
70
55
ZIC2
Scr
LOR-253
Veh Cd Veh Cd
35
25
Shh
Scr
LOR-253
Veh Cd Veh Cd
70
55
35
β-actin

Figure 3D

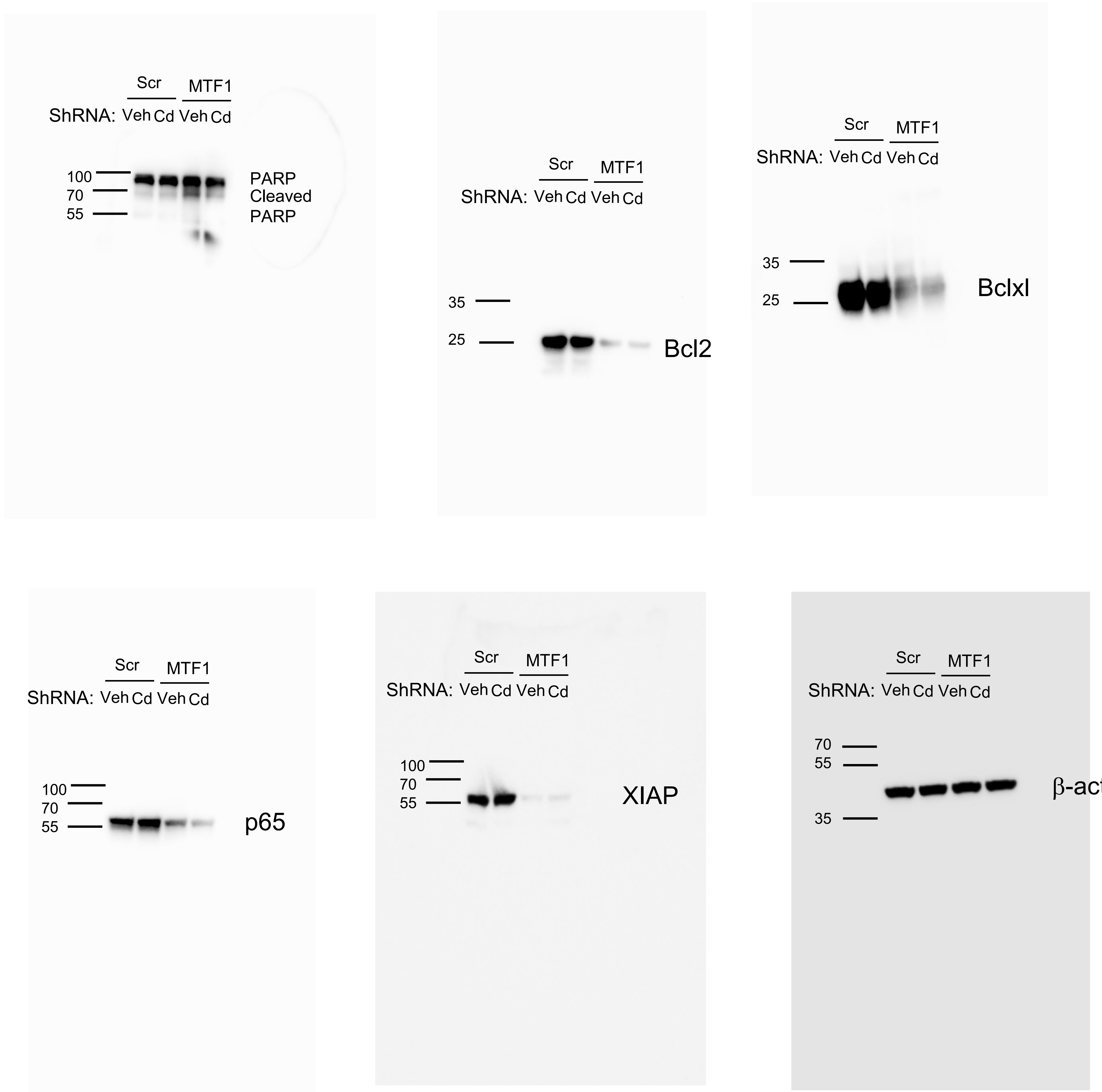
Scr
MTF1
ShRNA: Veh Cd Veh Cd
100
70
55
PARP
Cleaved
PARP
Scr
MTF1
ShRNA: Veh Cd Veh Cd
35
25
Bcl2
Scr
MTF1
ShRNA: Veh Cd Veh Cd
35
25
Bclxl
Scr
MTF1
ShRNA: Veh Cd Veh Cd
100
70
55
p65
Scr
MTF1
ShRNA: Veh Cd Veh Cd
100
70
55
XIAP
Scr
MTF1
ShRNA: Veh Cd Veh Cd
70
55
35
β-actin

Figure 3E

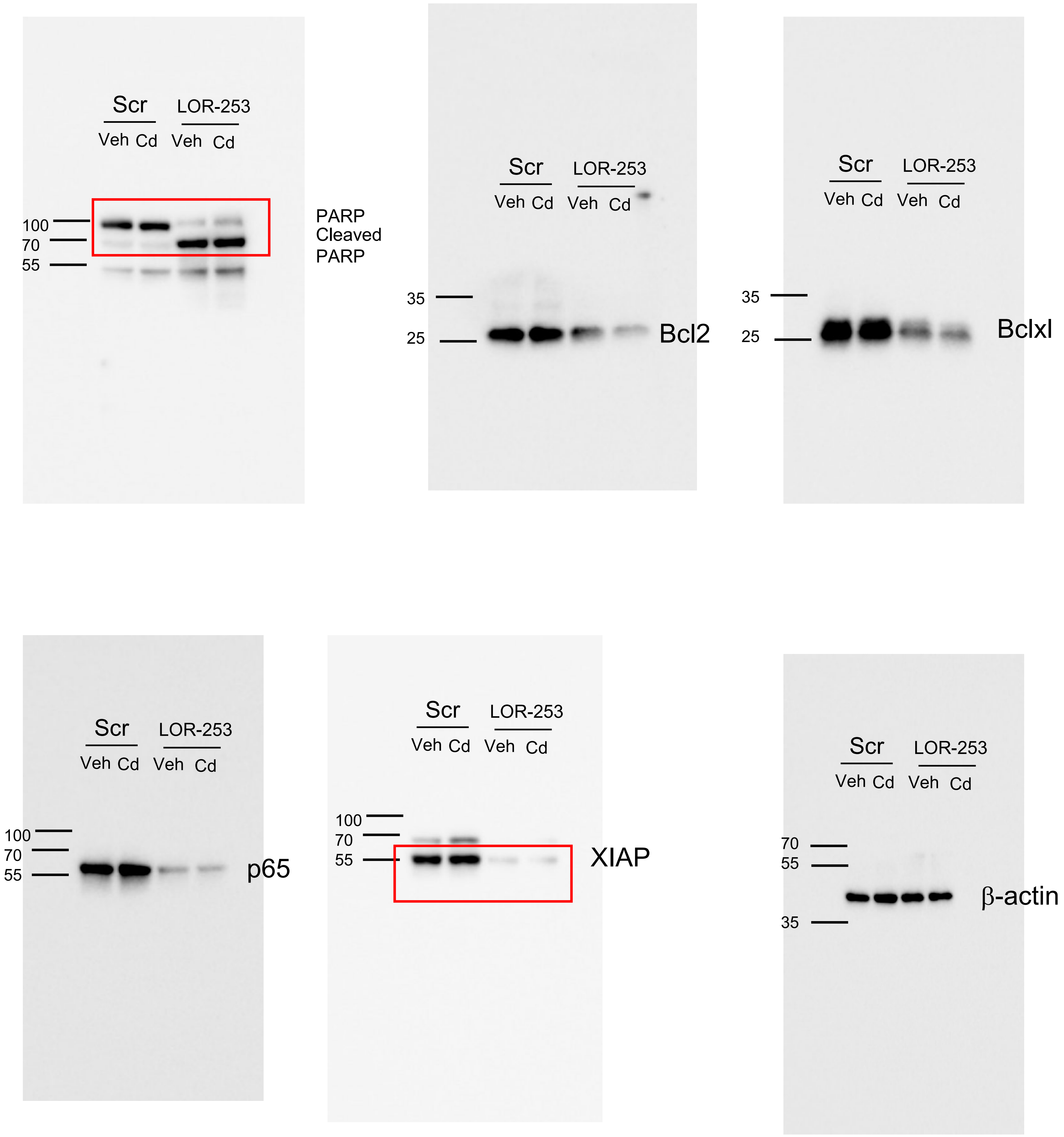

Scr
LOR-253
Veh Cd Veh Cd
100
70
55
PARP
Cleaved
PARP
35
25
Bcl2
Bclxl
p65
XIAP
β-actin

Figure 4A

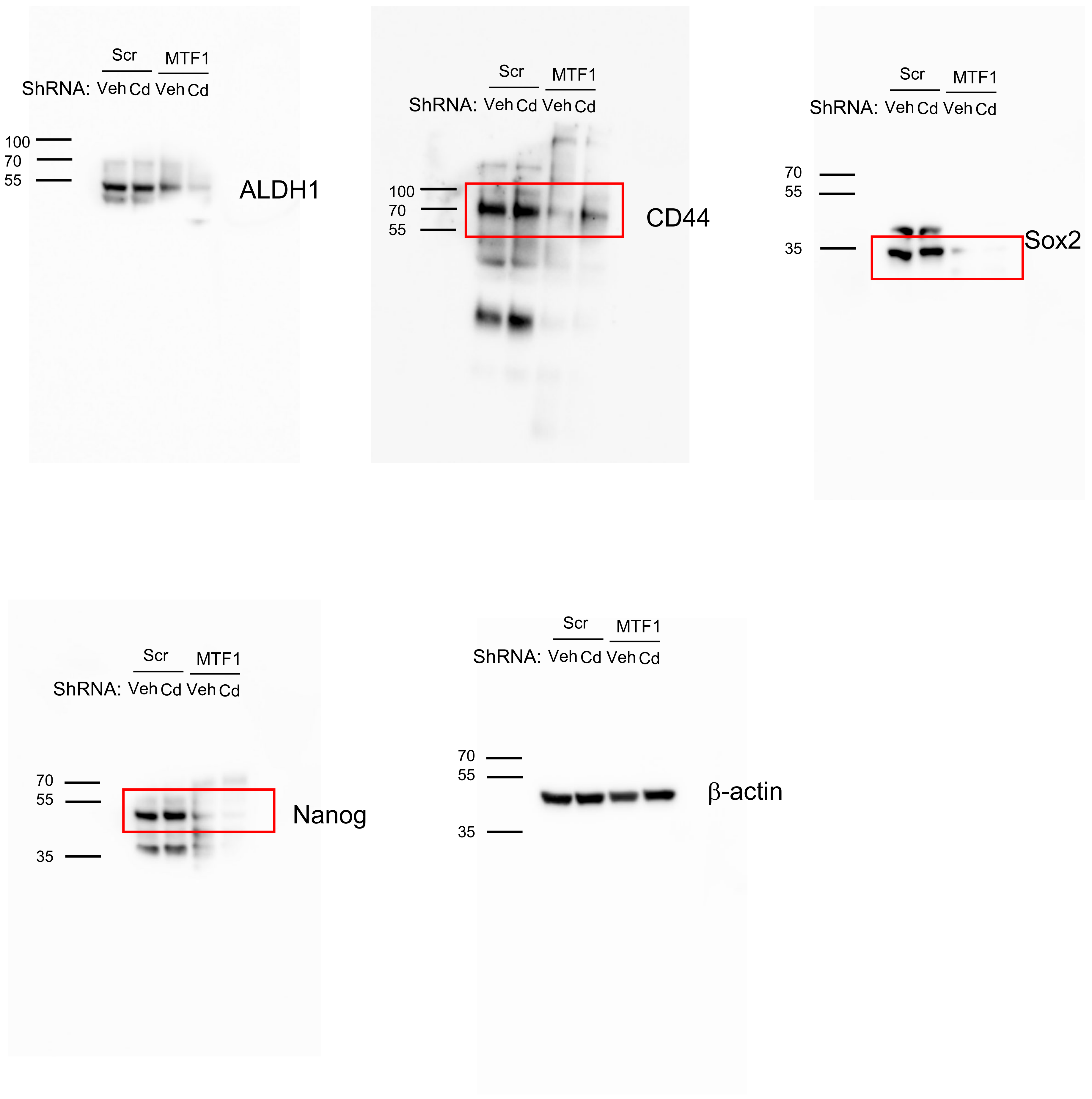

Scr
MTF1
ShRNA: Veh Cd Veh Cd
100
70
55
ALDH1
Scr
MTF1
ShRNA: Veh Cd Veh Cd
100
70
55
CD44
Scr
MTF1
ShRNA: Veh Cd Veh Cd
70
55
35
Sox2
Scr
MTF1
ShRNA: Veh Cd Veh Cd
70
55
35
Nanog
Scr
MTF1
ShRNA: Veh Cd Veh Cd
70
55
35
β-actin

Figure 4C

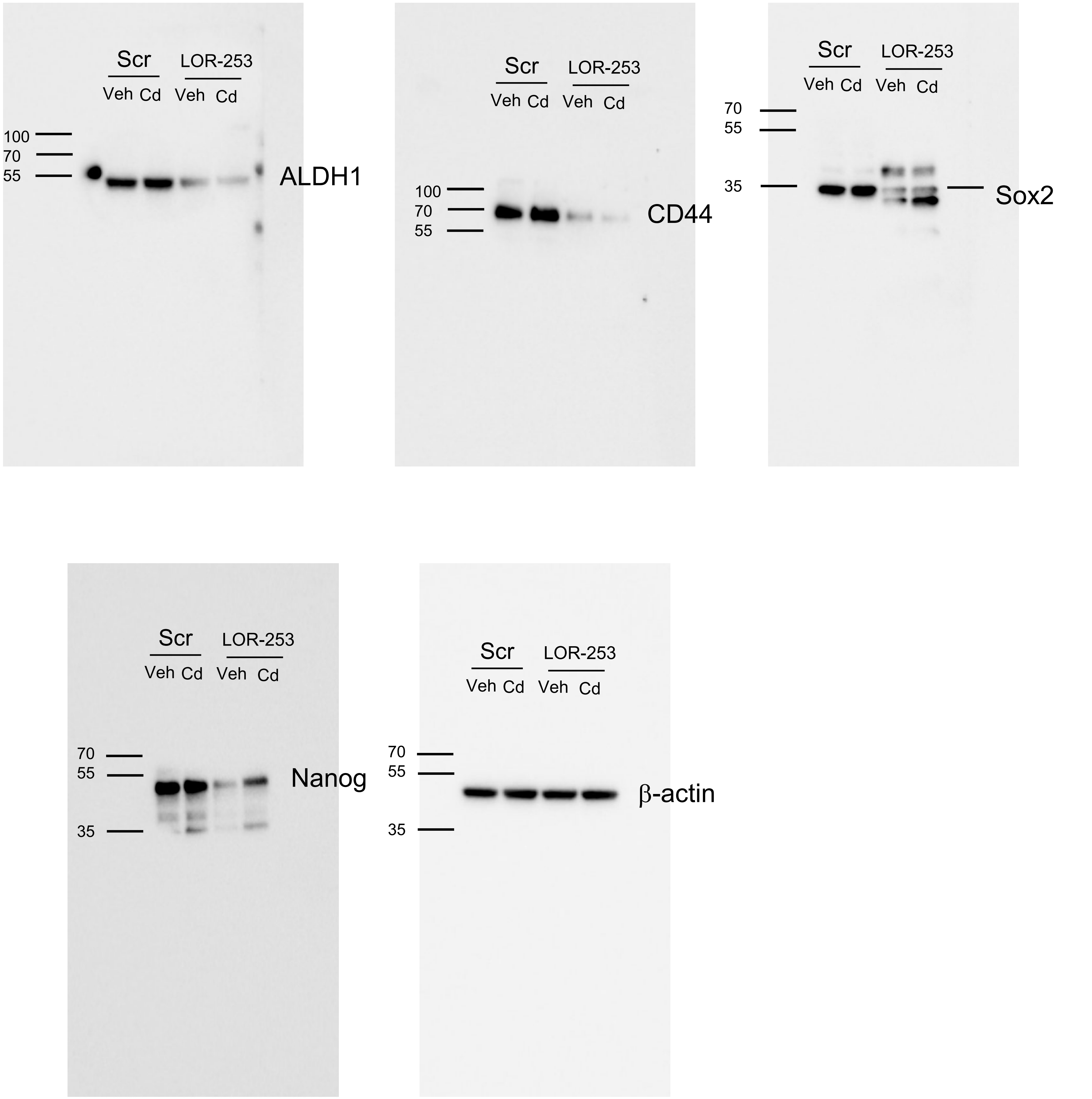

Scr
LOR-253
Veh
Cd
Veh
Cd
100
70
55
ALDH1
Scr
LOR-253
Veh
Cd
Veh
Cd
100
70
55
CD44
Scr
LOR-253
Veh
Cd
Veh
Cd
70
55
35
Sox2
Scr
LOR-253
Veh
Cd
Veh
Cd
70
55
35
Nanog
Scr
LOR-253
Veh
Cd
Veh
Cd
70
55
35
β-actin

Figure 4D

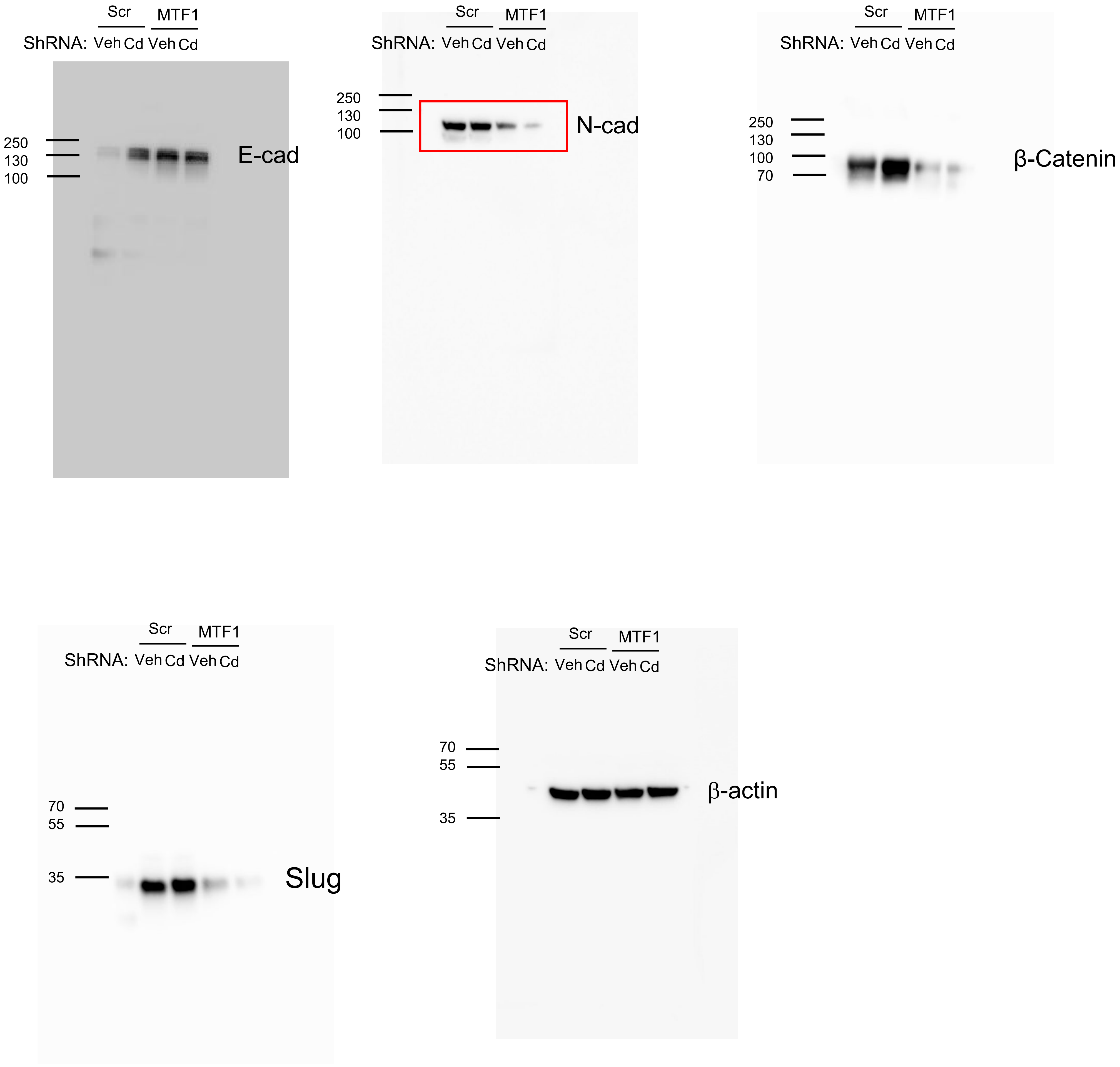
Scr
MTF1
ShRNA: Veh Cd Veh Cd
250
130
100
E-cad
Scr
MTF1
ShRNA: Veh Cd Veh Cd
250
130
100
N-cad
Scr
MTF1
ShRNA: Veh Cd Veh Cd
250
130
100
70
β-Catenin
Scr
MTF1
ShRNA: Veh Cd Veh Cd
70
55
35
Slug
Scr
MTF1
ShRNA: Veh Cd Veh Cd
70
55
35
β-actin

Figure 4E

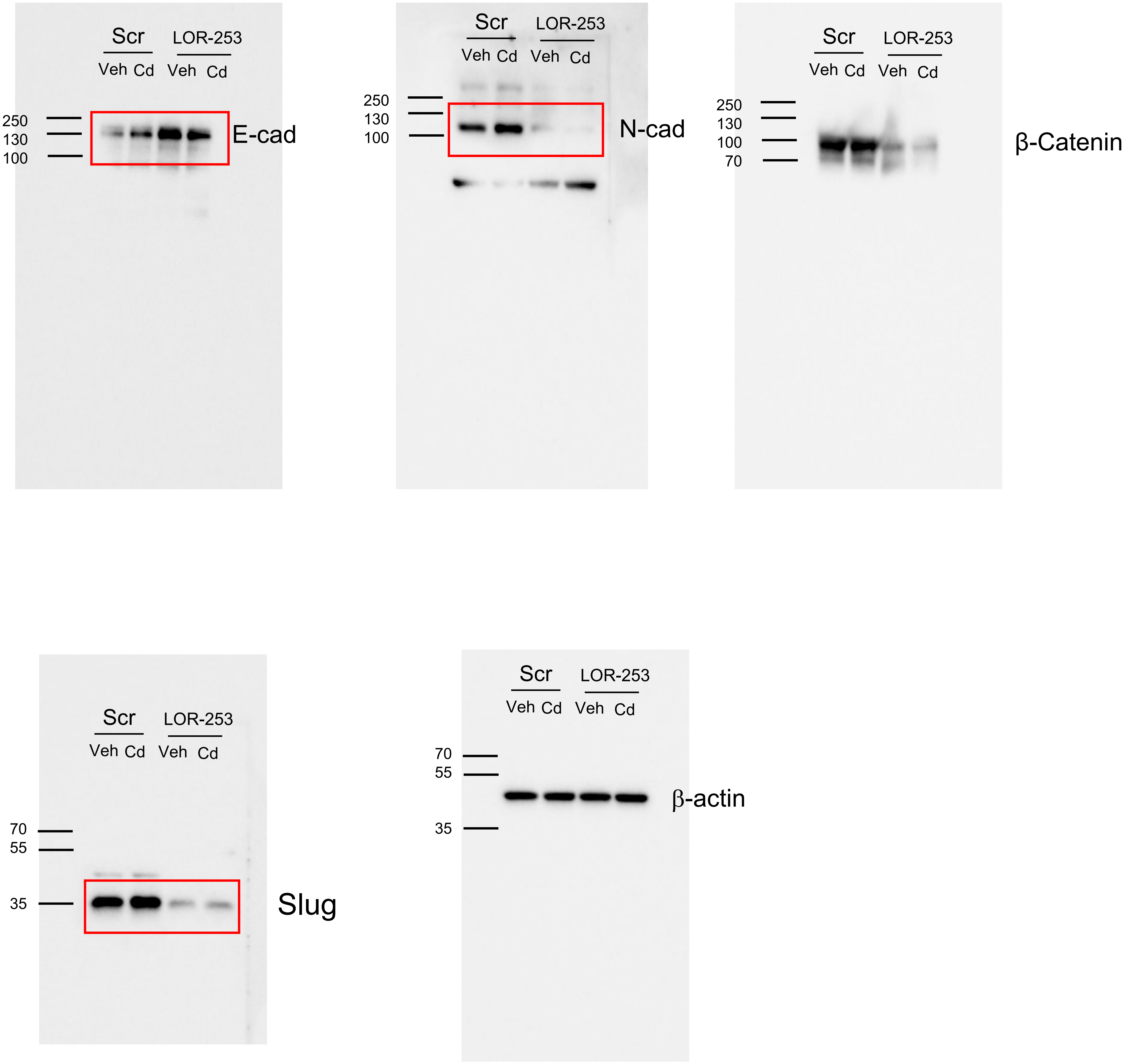

Scr
LOR-253
Veh Cd Veh Cd
250
130
100
E-cad
Scr
LOR-253
Veh Cd Veh Cd
250
130
100
N-cad
Scr
LOR-253
Veh Cd Veh Cd
250
130
100
70
β-Catenin
Scr
LOR-253
Veh Cd Veh Cd
70
55
35
Slug
Scr
LOR-253
Veh Cd Veh Cd
70
55
35
β-actin

Figure 4G

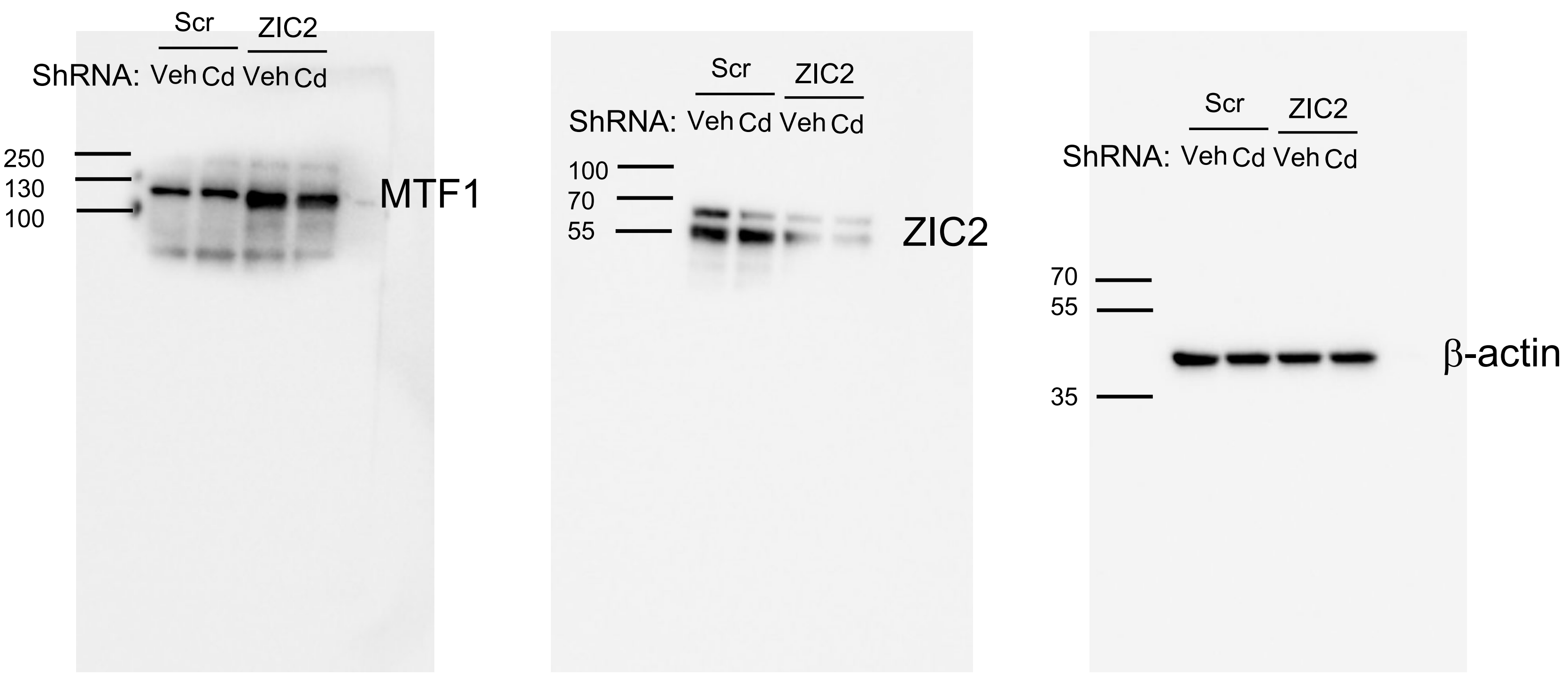

Scr
ZIC2
ShRNA: Veh Cd Veh Cd
250
130
100
MTF1
Scr
ZIC2
ShRNA: Veh Cd Veh Cd
100
70
55
ZIC2
Scr
ZIC2
ShRNA: Veh Cd Veh Cd
70
55
35
β-actin

Figure 5A

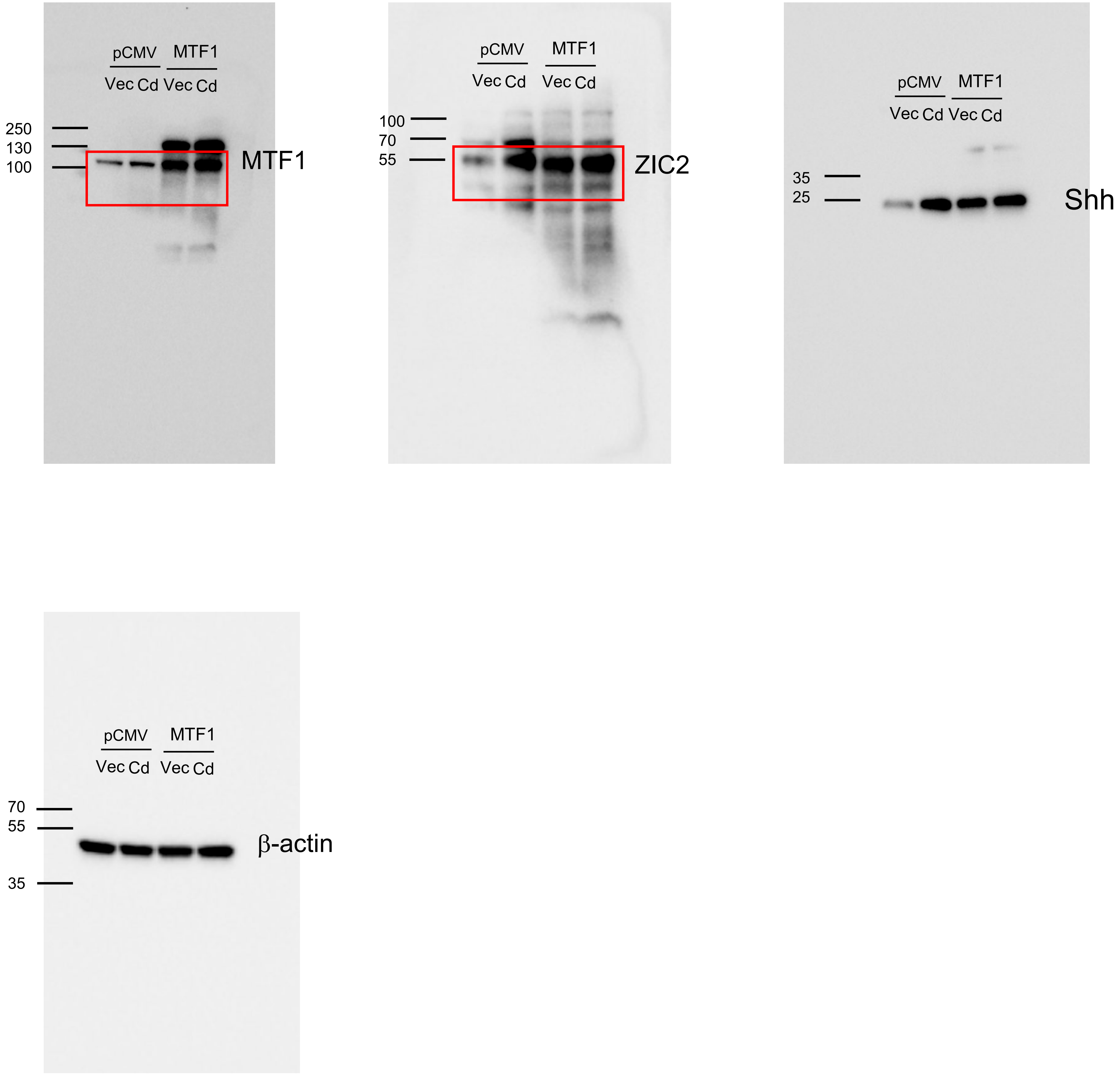
pCMV
MTF1
Vec Cd Vec Cd
250
130
100
MTF1
pCMV
MTF1
Vec Cd Vec Cd
100
70
55
ZIC2
pCMV
MTF1
Vec Cd Vec Cd
35
25
Shh
pCMV
MTF1
Vec Cd Vec Cd
70
55
35
β-actin

Figure 5B

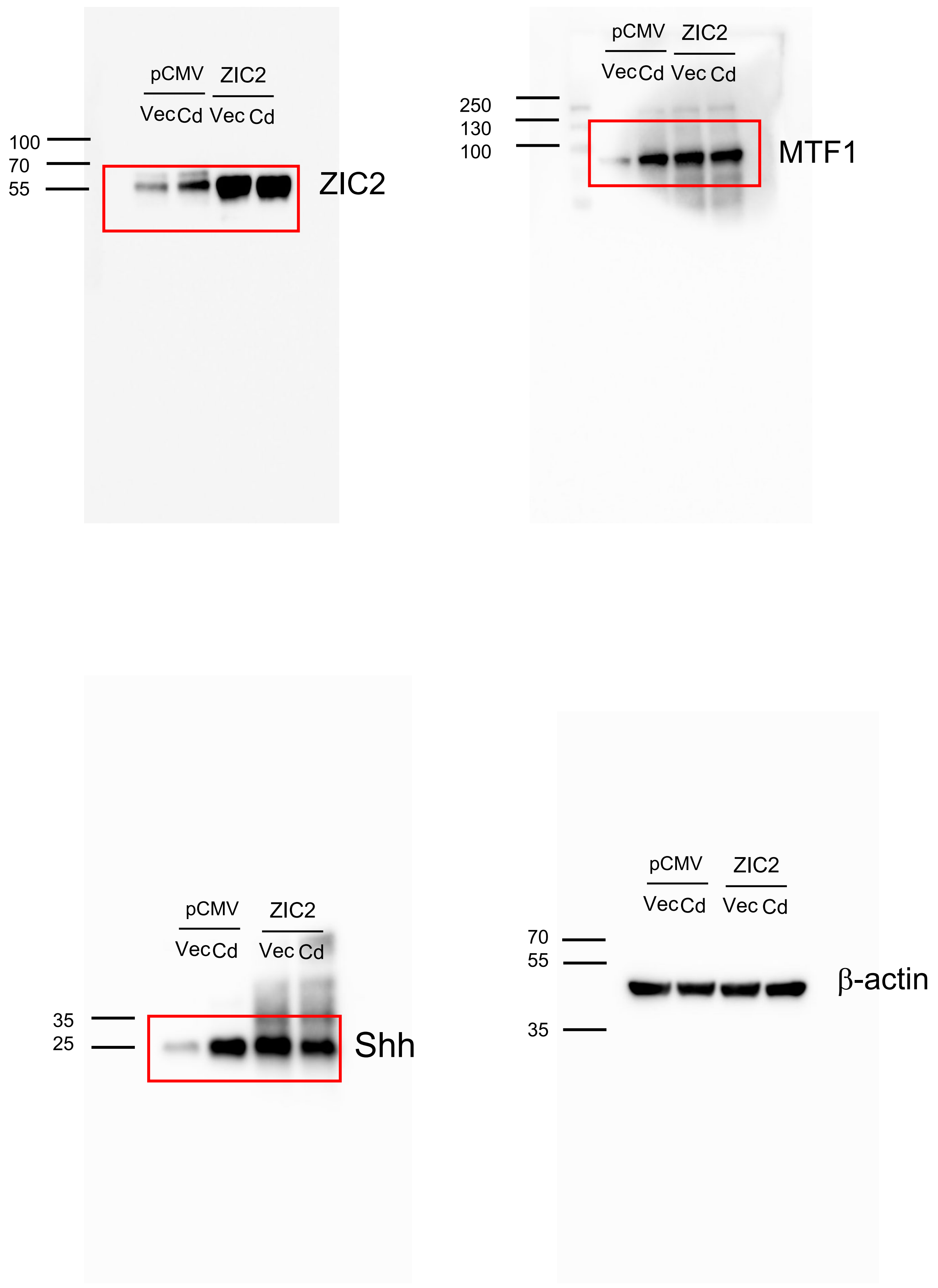
pCMV
ZIC2
Vec Cd Vec Cd
100
70
55
ZIC2
pCMV
ZIC2
Vec Cd Vec Cd
250
130
100
MTF1
pCMV
ZIC2
Vec Cd Vec Cd
35
25
Shh
pCMV
ZIC2
Vec Cd Vec Cd
70
55
35
β-actin

Figure 5C

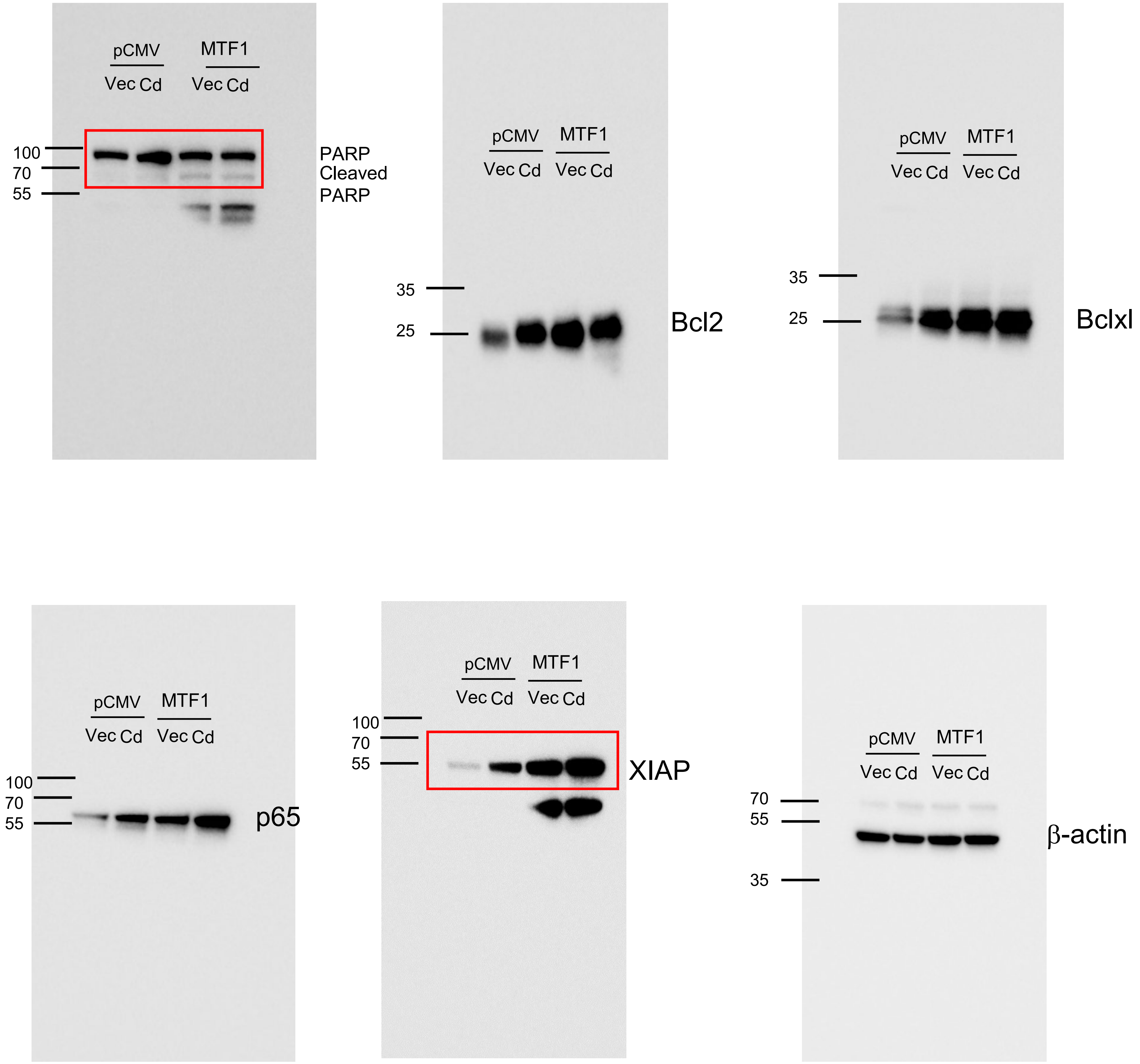
pCMV
MTF1
Vec Cd
Vec Cd
100
70
55
PARP
Cleaved
PARP
pCMV
MTF1
Vec Cd
Vec Cd
35
25
Bcl2
pCMV
MTF1
Vec Cd
Vec Cd
35
25
Bclxl
pCMV
MTF1
Vec Cd
Vec Cd
100
70
55
p65
pCMV
MTF1
Vec Cd
Vec Cd
100
70
55
XIAP
pCMV
MTF1
Vec Cd
Vec Cd
70
55
35
β-actin

Figure 5D

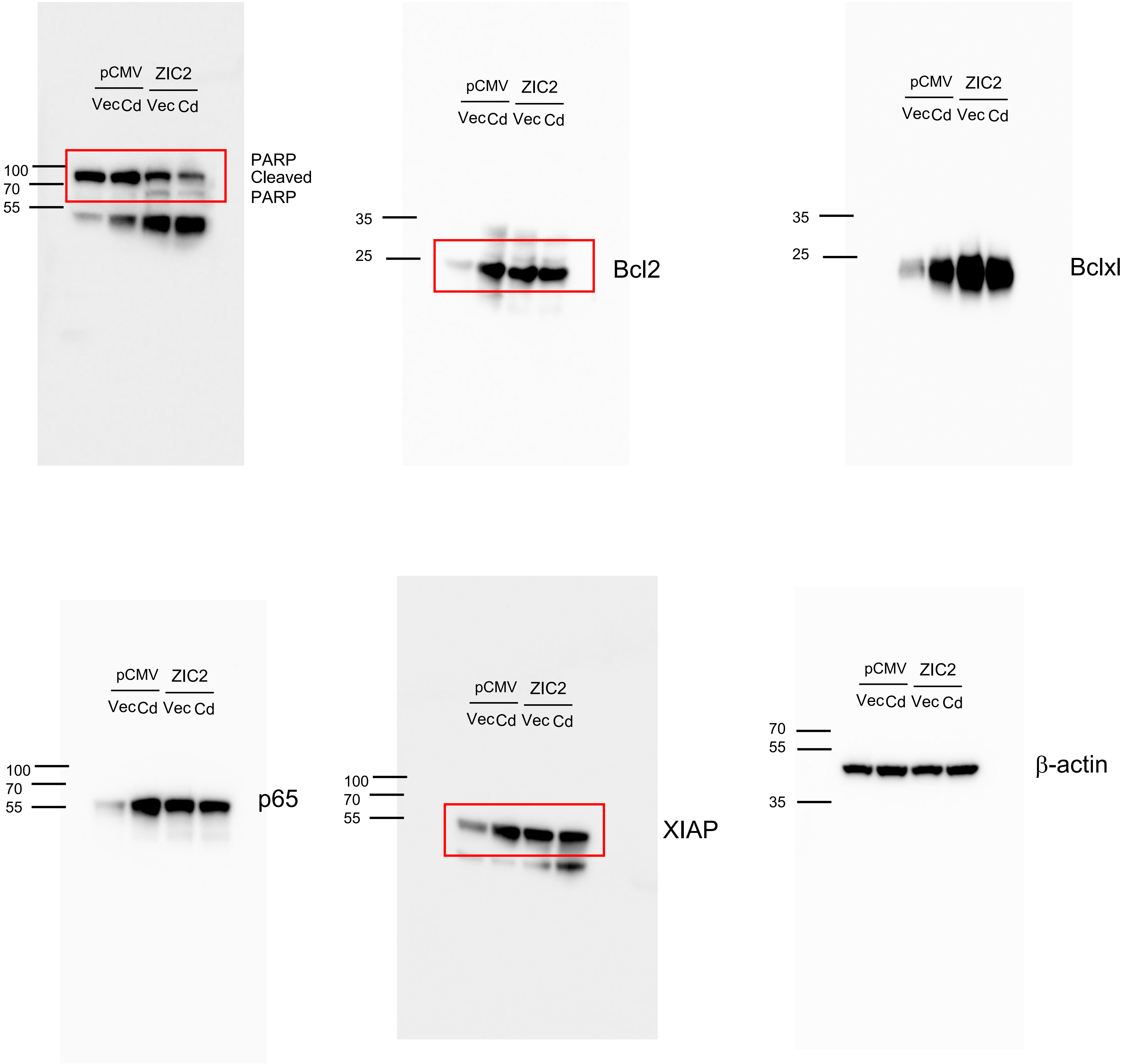
pCMV
ZIC2
Vec Cd Vec Cd
100
70
55
PARP
Cleaved
PARP
35
25
Bcl2
Bclxl
p65
XIAP
70
55
35
β-actin

Figure 6A

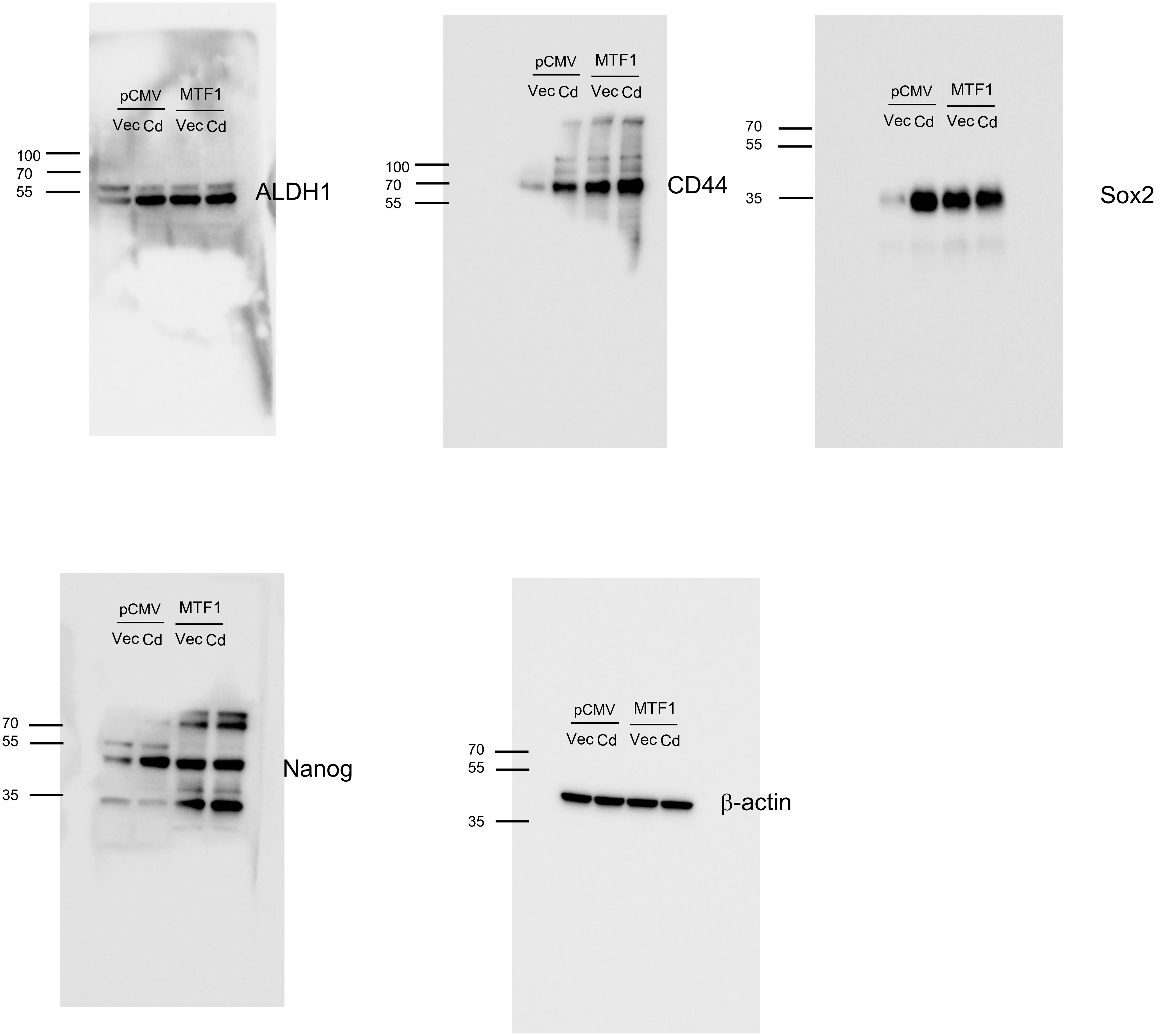
pCMV
MTF1
Vec Cd Vec Cd
100
70
55
ALDH1
pCMV
MTF1
Vec Cd Vec Cd
100
70
55
CD44
pCMV
MTF1
Vec Cd Vec Cd
70
55
35
Sox2
pCMV
MTF1
Vec Cd Vec Cd
70
55
35
Nanog
pCMV
MTF1
Vec Cd Vec Cd
70
55
35
β-actin

Figure 6C

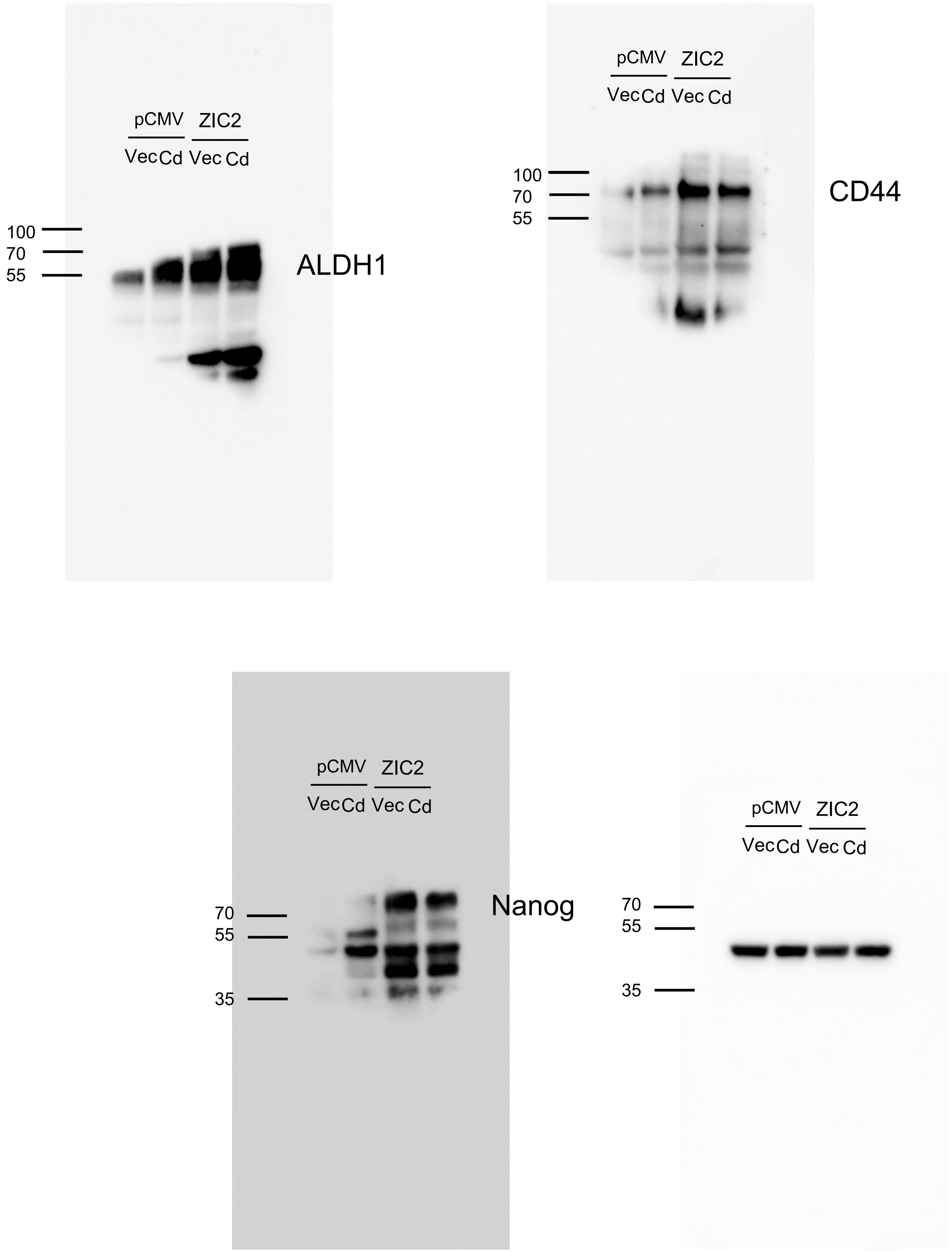
pCMV
ZIC2
Vec Cd Vec Cd
100
70
55
ALDH1
pCMV
ZIC2
Vec Cd Vec Cd
100
70
55
CD44
pCMV
ZIC2
Vec Cd Vec Cd
70
55
35
Nanog
pCMV
ZIC2
Vec Cd Vec Cd
70
55
35

Figure 6D

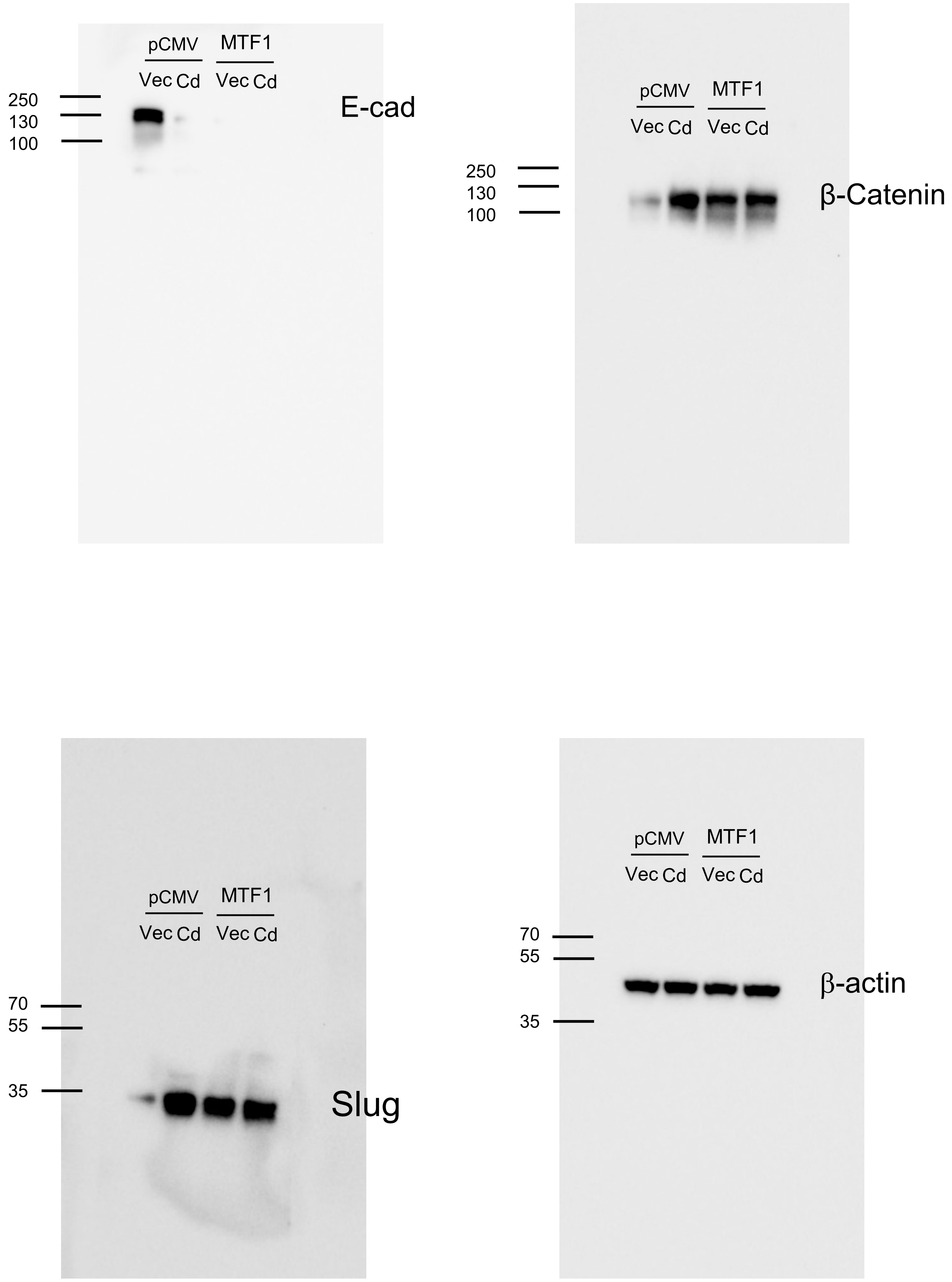

pCMV
MTF1
Vec Cd Vec Cd
250
130
100
E-cad
pCMV
MTF1
Vec Cd Vec Cd
250
130
100
β-Catenin
pCMV
MTF1
Vec Cd Vec Cd
70
55
35
Slug
pCMV
MTF1
Vec Cd Vec Cd
70
55
35
β-actin

Figure 6F

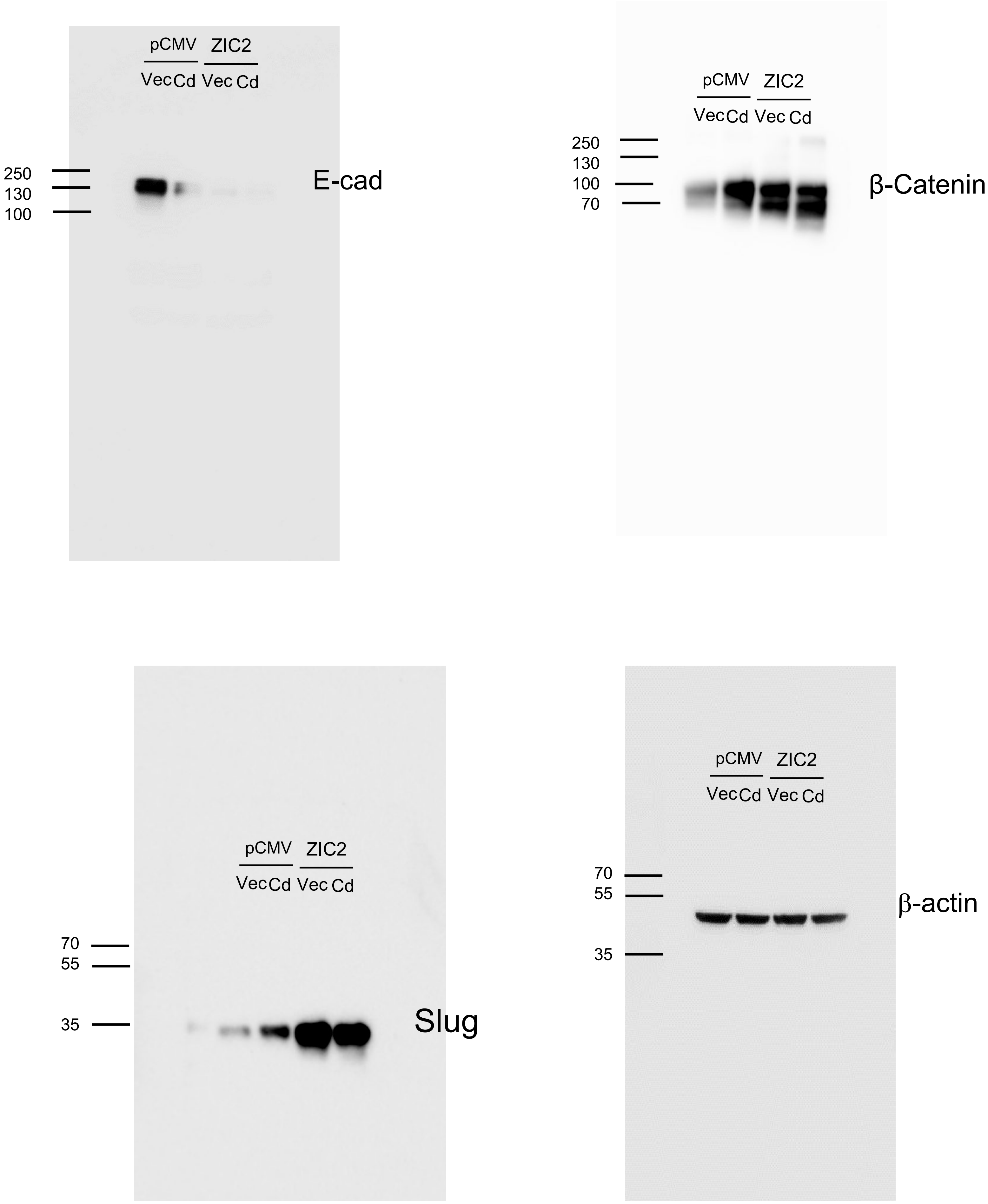
pCMV
ZIC2
Vec Cd Vec Cd
250
130
100
E-cad
pCMV
ZIC2
Vec Cd Vec Cd
250
130
100
70
β-Catenin
pCMV
ZIC2
Vec Cd Vec Cd
70
55
35
Slug
pCMV
ZIC2
Vec Cd Vec Cd
70
55
35
β-actin

Figure 7A

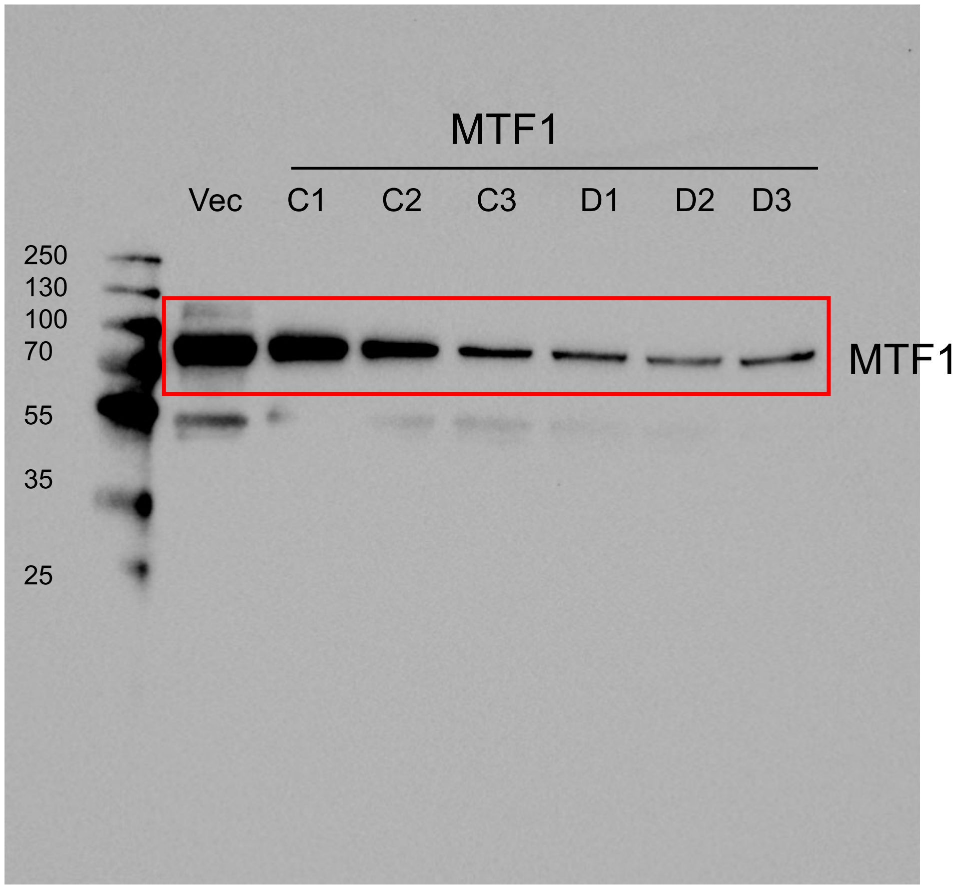

MTF1
Vec
C1
C2
C3
D1
D2
D3
250
130
100
70
55
35
25
MTF1

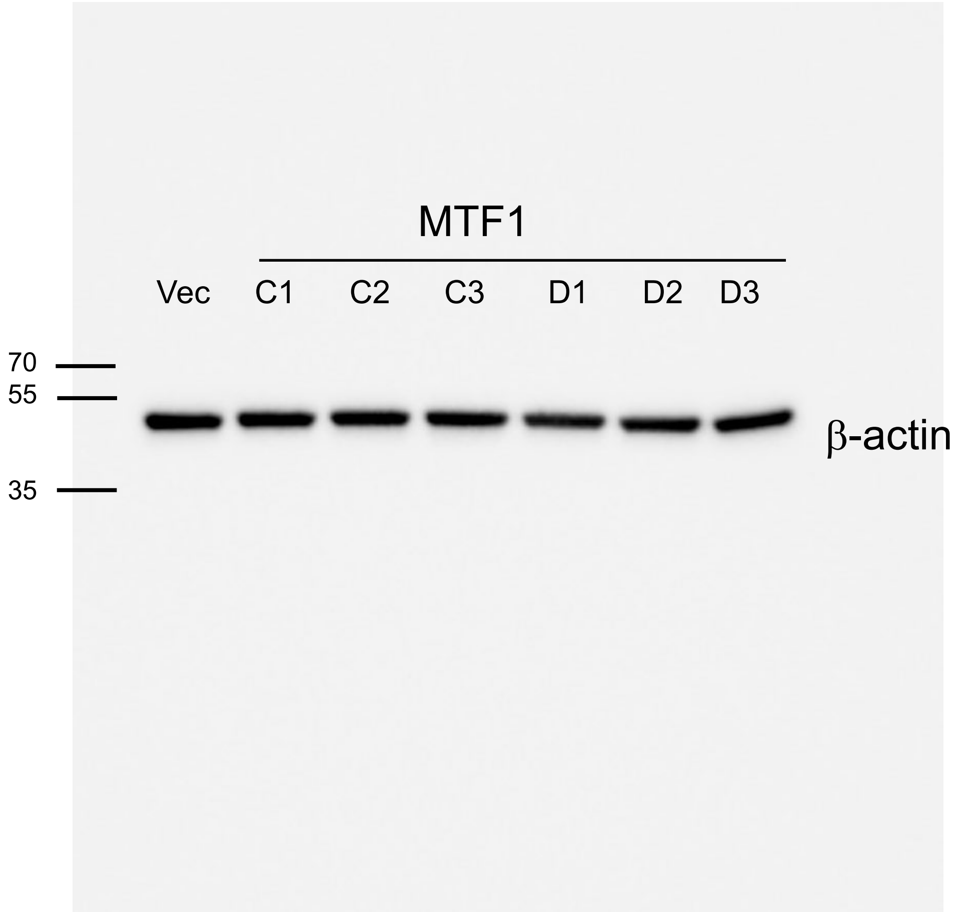

MTF1
Vec
C1
C2
C3
D1
D2
D3
70
55
35
β-actin

Figure 7B

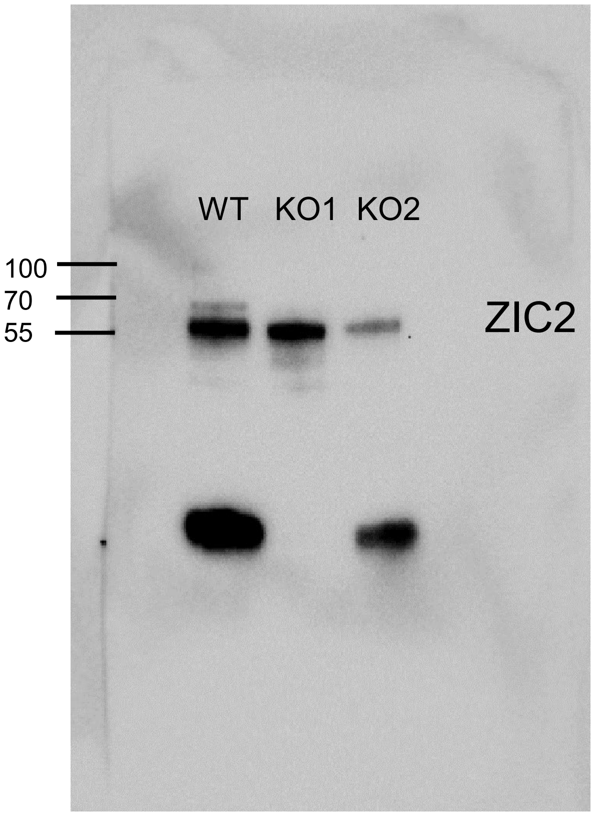

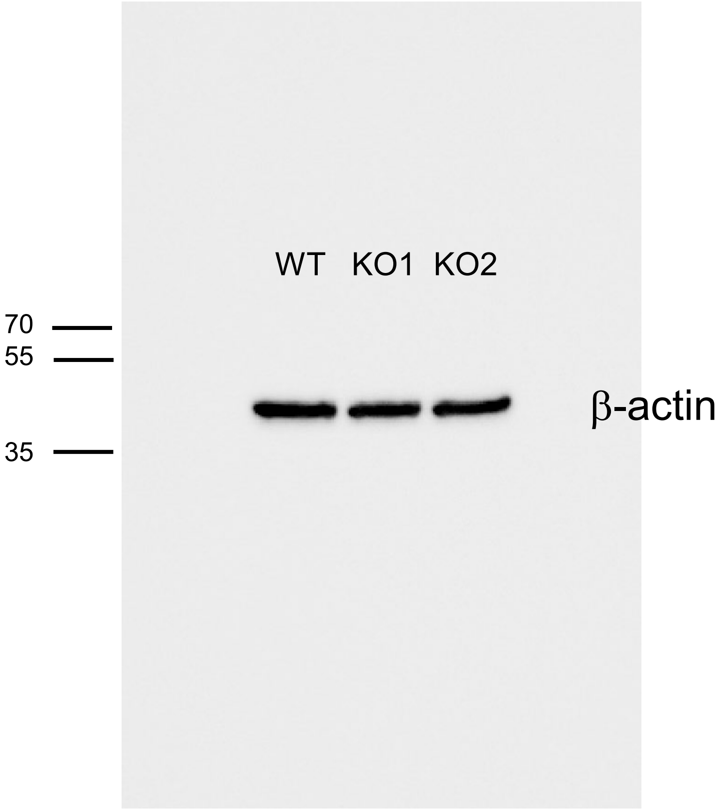

Supplement: Supplementary file 1 — Supplementary figures and tables. [file ijbsv21p3614s1.pdf]
